# Evaluating the Antiviral Efficacy of Encapsulated PKC Inhibitor BIM‐I against influenza A Virus Infection

**DOI:** 10.1002/adhm.202504060

**Published:** 2025-11-22

**Authors:** Laura Klement, Jana Ismail, Josefine Schroeder, Amod Godbole, Johanna Schreiber, Christine Weber, Zoltan Cseresnyes, Marc T. Figge, Bettina Löffler, Ulrich S. Schubert, Stephanie Schubert, Christina Ehrhardt, Carsten Hoffmann

**Affiliations:** ^1^ Institute of Molecular Cell Biology Center for Molecular Biomedicine (CMB) University Hospital Jena Friedrich Schiller University Jena Jena Germany; ^2^ Laboratory of Organic and Macromolecular Chemistry (IOMC) Friedrich Schiller University Jena Jena Germany; ^3^ Section of Experimental Virology Institute of Medical Microbiology Center for Molecular Biomedicine (CMB) University Hospital Jena Jena Germany; ^4^ Applied Systems Biology Leibniz Institute for Natural Product Research and Infection Biology Hans Knöll Institute (HKI) Jena German; ^5^ Institute of Medical Microbiology University Hospital Jena Jena Germany; ^6^ Jena Center for Soft Matter (JCSM) Friedrich Schiller University Jena Jena Germany; ^7^ Institute of Microbiology Faculty of Biological Sciences Friedrich Schiller University Jena Jena Germany

**Keywords:** antivirals, bisindolylmaleimide‐I (BIM‐I), influenza A virus (IAV), nanoparticles (NPs), poly(2‐ethyl‐2‐oxazoline) (PEtOx), poly(lactic‐*co*‐glycolic acid) (PLGA), protein kinase C (PKC)

## Abstract

Influenza A virus (IAV) infections remain a major global health threat, as current vaccines and antivirals often lose efficacy due to frequent viral mutation and resistance development. This underscores the urgent need for novel therapeutic strategies, such as targeting host factors, which may reduce the likelihood of resistance. Here, we evaluated inhibitors of G protein‐coupled receptor kinases (GRKs; paroxetine, CMPD101) and protein kinase C (PKC; Gö6983, bisindolylmaleimide‐I (BIM‐I)) for anti‐IAV activity. GRK inhibition showed no significant effect, whereas PKC inhibition, particularly with BIM‐I, significantly reduced infection. To overcome BIM‐I's poor solubility and concentration‐dependent cytotoxicity, it is encapsulated into poly(lactic‐*co*‐glycolic acid) (PLGA)‐based nanoparticles. To enhance nanoparticle performance, stealth polymers like polyethylene glycol (PEG) are commonly incorporated. However, concerns about PEG immunogenicity have increased interest in alternatives like poly(2‐ethyl‐2‐oxazoline) (PEtOx). We formulated BIM‐I‐loaded nanoparticles containing either PEG or PEtOx and characterized them for their physicochemical properties, cytotoxicity, antiviral efficacy, and cellular uptake. Encapsulation improved the cellular tolerability of BIM‐I while preserving its antiviral activity. Confocal microscopy confirmed efficient uptake of all formulations, particularly PEGylated and PEtOxylated nanoparticles. These findings highlight nanoparticle‐mediated delivery of BIM‐I as a promising host‐directed antiviral strategy against IAV and support PEtOx as a viable PEG alternative in nanomedicine.

## Graphical abstract

This study explores nanoparticle delivery of the protein kinase C inhibitor bisindolylmaleimide‐I (BIM‐I) to combat influenza A virus infections. Encapsulation in biodegradable PLGA nanoparticles improved safety while maintaining the compound's strong antiviral activity. Surface modification with PEG or PEtOx enhanced cellular uptake of the nanoparticles, highlighting a promising host‐directed nanomedicine approach and supporting PEtOx as a viable alternative to PEG.

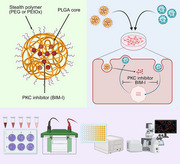

## 1 Introduction

Respiratory viruses remain a significant threat to global public health, with influenza A viruses (IAVs) representing one of the most prominent and persistent challenges. Seasonal influenza epidemics result in approximately one billion annual cases, including three to five million cases of severe illness and up to 650 000 deaths per year, causing substantial morbidity, mortality, and economic costs [1]. Addressing severe IAV infections remains an unresolved challenge since antiviral treatment options are limited. The constant evolution of IAVs, driven by antigenic drift and shift, contributes to seasonal epidemics, increases the risk of epidemics and pandemics, and impedes the development of effective drugs and vaccines. Vaccination remains the most effective protection against IAV epidemics, despite time‐consuming production, the requirement for annual reformulation, and variable efficacy against emerging subtypes [1].

At present, three classes of antiviral medications are approved by the European Medicines Agency (EMA) and the Food and Drug Administration (FDA) for IAV infection treatment: matrix protein 2 (M2) ion channel inhibitors (amantadine, rimantadine), neuraminidase inhibitors (NAIs) (zanamivir, oseltamivir, peramivir), and the polymerase acidic endonuclease inhibitor baloxavir marboxil. However, most circulating IAVs are now resistant to M2 ion channel inhibitors, and resistance to certain NAIs has emerged, highlighting the urgent need to develop novel antiviral treatment strategies [2]. Since viruses rely on the host cell machinery for replication, targeting essential host factors presents a promising approach for antiviral therapy. Given the dependence of viruses on cellular kinases, kinase inhibitors offer potential therapeutic options, as disrupting these pathways may be challenging for the virus to evade [3]

To date, approximately 28 different cellular kinases have been identified to play a role in influenza virus infection [3]. Among them, G protein‐coupled receptor (GPCR) kinases (GRKs) were reported to be involved in different viral infections [4]. These serine‐threonine kinases are not only primarily known to phosphorylate GPCRs for their desensitization but also regulate other cellular proteins involved in cell proliferation, survival, apoptosis, or immunity. Of the seven GRK isoforms, only GRK2, 3, 5, and 6 are ubiquitously expressed in humans [5, 6, 7]. Specifically, the isoform GRK2 has been shown to play a role in the yellow fever virus entry, the RNA synthesis of the dengue virus, and the infection of the hepatitis C virus (HCV) [4, 8]. Moreover, GRK2 downregulation was shown to inhibit IAV infection [9]. In 2018, Yanguez et al. reported that GRK2 is phosphorylated in the early stages of IAV infection and that it is essential for viral uncoating. By treatment with paroxetine, the authors were able to decrease IAV infection in human lung cells and in a mouse model [10]. Paroxetine is a clinically approved selective serotonin reuptake inhibitor (SSRI) and has been demonstrated to inhibit GRK2 activity in vitro, in cell culture, and in mice [11, 12]. If paroxetine demonstrates efficacy against IAVs, it could be repurposed as a potential antiviral therapeutic agent. The repurposing of approved drugs with established safety and favorable pharmacokinetics accelerates the development of novel therapeutic strategies while reducing costs and minimizing the risk of adverse effects [13]

Furthermore, the serine‐threonine kinase protein kinase C (PKC) was implicated to mediate different steps of the influenza virus life cycle [3]. At least ten human PKC isoforms are expressed in many different tissues and regulate various physiological processes, such as cell proliferation, differentiation, migration, survival, and apoptosis [14, 15]. Among them, the isoform PKCβII was shown to mediate IAV entry via inhibition of virus release from the late endosome [16]. PKCδ was identified to control ribonucleoprotein assembly for subsequent viral genome replication by phospho‐regulating nucleoprotein oligomerization [17, 18]. Several studies have reported that PKC phosphorylates different viral proteins, including matrix protein 1, RNA‐dependent RNA polymerase subunit polymerase basic 1, nonstructural protein, and nucleoprotein, thereby mediating successful infection [17, 19, 20]. Furthermore, the highly selective PKC inhibitor bisindolylmaleimide‐I (BIM‐I) has been shown to inhibit IAV infection [20, 21]. BIM‐I, also known as Gö6850 or GF109203X, inhibits the PKC isoforms α, βI, βII, γ, δ, and ε by competitive binding at the ATP‐binding site [22, 23]. It has been shown to exhibit anticancer activity via different pathways [24]. and to reduce severe acute respiratory syndrome coronavirus 2 (SARS‐CoV‐2) infection [25] highlighting its potential as a drug candidate. However, BIM‐I has been reported to directly inhibit the human ether‐à‐go‐go‐related gene potassium channels, thereby interfering with cardiac action potentials [26, 27]. Additionally, the compound's hydrophobic nature limits its suitability for in vivo use unless formulated with appropriate delivery strategies to improve drug solubility and bioavailability. Furthermore, the short half‐life and broad systemic distribution of conventionally administered drugs necessitate high dosages, increasing the risk of adverse effects. However, utilizing advanced delivery systems, such as encapsulation in nanoparticles (NPs), can overcome these limitations [28].

Poly(lactic‐*co*‐glycolic acid) (PLGA) is one of the most widely used and well‐characterized polymers approved by the FDA and EMA for drug delivery applications. Its biocompatibility, biodegradability, and suitability for targeted delivery make it an attractive platform for pharmaceutical formulations, in particular in the form of nano‐ and microparticles [29]. Upon administration, PLGA is hydrolyzed into the endogenously occurring metabolic byproducts lactic acid and glycolic acid, which are metabolized via the Krebs cycle and eliminated as carbon dioxide (CO_2_) and water, reducing the risk of long‐term accumulation [30]. PLGA‐based drug delivery systems can be tailored for controlled and prolonged drug release, ensuring that therapeutic levels are maintained while reducing dosing frequency and decreasing the risk of side effects [31]. However, their clinical application is limited due to rapid opsonization of hydrophobic particles and clearance by the reticulo‐endothelial system following intravenous administration [32].

To overcome these limitations, polyethylene glycol (PEG)‐functionalized PLGA NPs are often employed in drug delivery systems [30, 32]. PEGylation creates a hydrophilic “stealth” layer around the particles, reducing aggregation, opsonization, and elimination by the immune system, thereby prolonging circulation time and enhancing drug delivery [33]. PEG is commonly used in a wide range of consumer products, including cosmetics like shampoos, toothpaste, or soaps. However, repeated exposure to PEG can induce the production of anti‐PEG antibodies, which may lead to hypersensitivity reactions ranging from mild allergic responses to severe anaphylaxis, a phenomenon known as PEG immunogenicity [34, 35]. Screening of blood plasma samples from healthy donors revealed that anti‐PEG antibodies were present in approximately 72 to 83% of the population [36, 37]. This creates an urgent need for alternative stealth polymers that can avoid triggering immune responses. Promising candidates are poly(2‐oxazoline)s (POx), specifically poly(2‐ethyl‐2‐oxazoline) (PEtOx), which exhibits comparable stealth properties, hydrophilicity, and biocompatibility [38, 39, 40]. Although PEtOx has been successfully used in other nanocarrier systems, to the best of our knowledge, POxylation of PLGA NPs by formulation of block copolymers composed of PEtOx and PLGA has so far only been reported by us. In particular, Dirauf et al. compared PEtOx‐*b*‐PLGA block copolymers with the commercially available PEG‐*b*‐PLGA, demonstrating comparable physicochemical properties [39]. However, until now, a comprehensive evaluation of cytocompatibility, drug encapsulation efficiency, and biological performance in the context of drug delivery has not yet been conducted.

In this study, we investigated the role of host kinase modulation as a strategy to interfere with IAV replication. Therefore, we assessed the effect of different GRK and PKC inhibitors on IAV replication in cell culture. While the GRK inhibitors paroxetine and CMPD101 had no effect on viral replication in A549 cells, the PKC inhibitors Gö6983 and BIM‐I significantly reduced viral titers and viral protein expression. To overcome BIM‐I's limited solubility and cytotoxicity, the compound was encapsulated into PLGA‐based NPs, achieving improved cellular tolerability while maintaining the antiviral efficacy. Furthermore, to enhance the clinical applicability of the NPs, the stealth polymers PEG and its emerging alternative PEtOx were incorporated. These modifications improved antiviral efficacy and NP uptake in different cellular systems, highlighting their potential as an antiviral treatment strategy.

## 2 Results and Discussion

### 2.1 PKC Inhibitors Significantly Reduce IAV Infection in A549 Cells, Whereas GRK Inhibitors Show no Antiviral Effect

**FIGURE 1:**
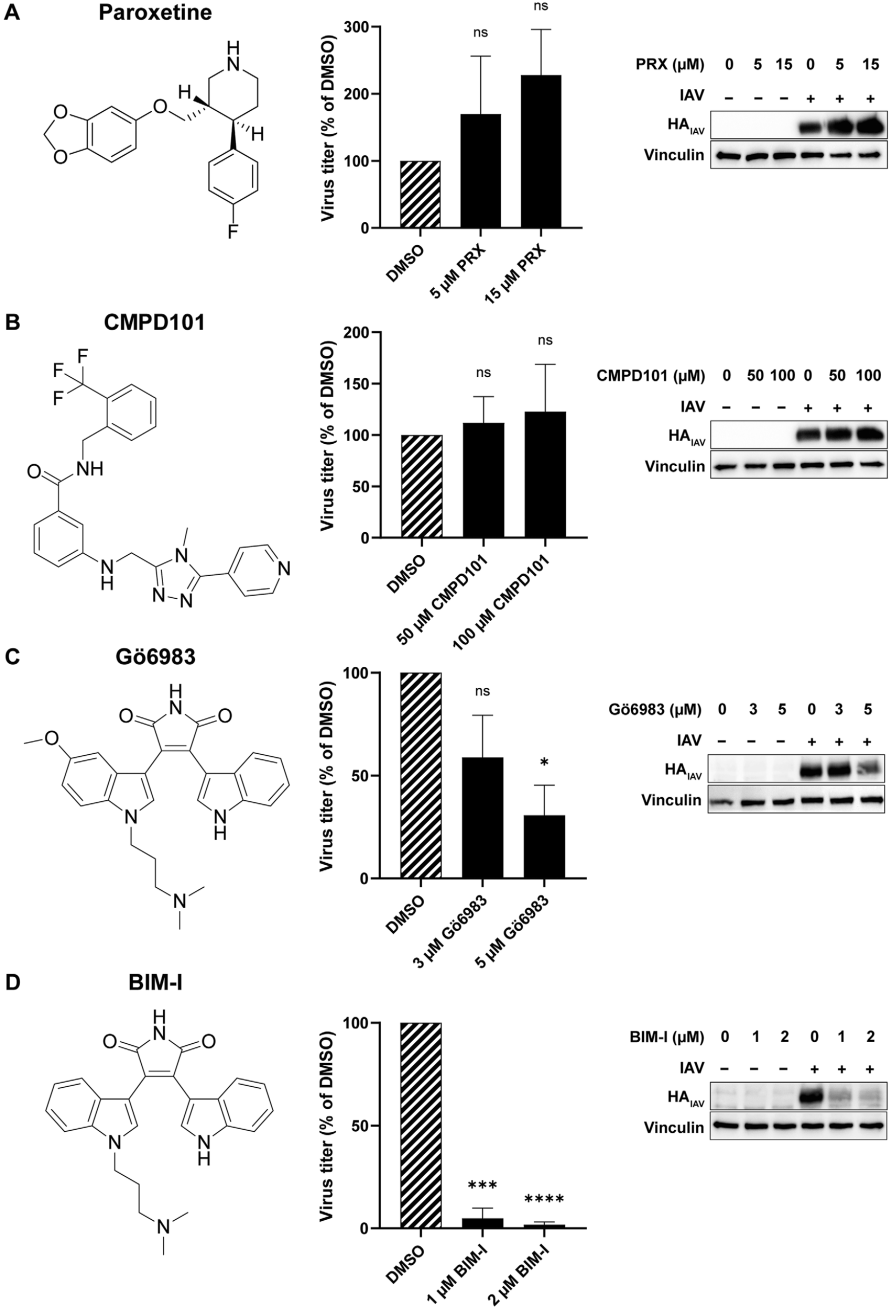
PKC inhibitors significantly reduce IAV infection in A549 cells, whereas GRK inhibitors show no antiviral effect. A‐D) A549 cells were infected with the IAV strain A/PR8 (H1N1) (IAV) at an MOI of 0.1 for 30 min. Subsequently, the virus was removed, and the cells were incubated with the indicated concentrations of paroxetine (PRX) (A), CMPD101 (B), Gö6983 (C), BIM‐I (D) or DMSO as the respective solvent control for a total of 24 h. Viral titers were determined by plaque assay, and the expression of the viral protein hemagglutinin (HA_IAV_) was assessed by Western blot analysis. Virus titers were normalized to DMSO, which was set to 100%. The mean + SD of n=3 independent experiments, each with two biological replicates for the viral titers, are shown. Statistical significance was determined using one sample t‐test comparing to 100. *p<0.05, ***p<0.001, ****p<0.0001.

Given the proposed roles of different GRK and PKC isoforms during IAV infection, we investigated the effects of four different small‐molecule kinase inhibitors, namely paroxetine, CMPD101, Gö6983, and BIM‐I, on IAV replication in a multi‐cycle viral growth experiment in A549 cells. A549 cells are human alveolar basal epithelial cells derived from a lung adenocarcinoma and serve as a model for respiratory virus infection experiments and antiviral drug testing [41]. Cells were infected with the IAV strain H1N1 A/Puerto Rico/8/1934 (A/PR8 (H1N1)) for 30 min, followed by incubation with the respective inhibitors at indicated concentrations or with DMSO as a solvent control for 24 h. Viral titers were quantified using plaque assays, while viral hemagglutinin (HA_IAV_) protein expression was assessed through Western blot analysis (Figure 1). Furthermore, possible cytotoxic effects of the different kinase inhibitors were evaluated in A549 cells after 24 h incubation, utilizing the CellTiter‐Blue Cell Viability Assay (Figure S1A‐D). This assay analyzes the metabolic capacity of viable cells by detecting their ability to reduce the agent resazurin to fluorescent resorufin.

To investigate the role of GRK2 in IAV infection, we first evaluated the effect of the GRK2 inhibitor paroxetine, which was previously reported to reduce IAV replication [10]. However, in our experimental setup, treatment with 5 or 15 µM of paroxetine did not significantly reduce IAV infection, as assessed by plaque assay and Western blot analysis of HA_IAV_ expression (Figure 1A). While previous studies utilized 50 µM paroxetine to achieve antiviral activity, this concentration was not feasible in our assay since it displayed significant cytotoxic effects in A549 cells, as observed with the CellTiter‐Blue Cell Viability Assay (Figure S1A).

Next, we tested CMPD101, a highly selective GRK2/3 inhibitor [42, 43], to further assess the role of GRK2 in IAV infection. However, treatment with 50 or 100 µM of CMPD101 did not significantly alter viral titers or HA_IAV_ protein expression compared to the DMSO control (Figure 1B), without impairing cell viability (Figure S1B). The effectiveness of the GRK inhibitor concentrations employed in A549 cells was validated through our recently published bioluminescence resonance energy transfer (BRET) assay (Figure S1E,F) [44]. Here, the recruitment of β‐arrestin2 to the β2 adrenergic receptor (β2AR) is detected in cells transfected with β‐arrestin2‐NanoLuciferase and β2AR‐Halo‐Tag. Upon stimulation of the β2AR with its ligand Iso, GRKs phosphorylate the receptor, mediating β‐arrestin2 recruitment to the receptor. In close proximity, energy is transferred from the donor NanoLuciferase to the acceptor Halo‐Tag, producing a measurable BRET signal, thereby enabling analysis of protein‐protein interaction (Figure S1E). Inhibition of GRKs by paroxetine and CMPD101 led to a concentration‐dependent decrease in the BRET fold change, since receptor phosphorylation by GRKs is inhibited, ultimately reducing β‐arrestin2 recruitment (Figure S1F). These findings suggest that GRK2 inhibition, via paroxetine or CMPD101, alone may not be sufficient to impair IAV replication under the tested conditions, although the inhibitors reach effective concentrations.

To investigate the role of PKC in IAV infection, we evaluated the structurally related PKC inhibitors Gö6983 and BIM‐I (also known as Gö6850 or GF109203X), which share the same core structure and differ only by a methoxy group (Figure 1C,D). Treatment with 5 µM Gö6983 significantly reduced viral titers and viral HA_IAV_ expression, indicating an inhibitory effect on IAV replication (Figure 1C), without impairing cell viability (Figure S1C). Administration of BIM‐I at concentrations of 1 µM and 2 µM resulted in a more pronounced antiviral effect, as evidenced by a stronger reduction in IAV infection, without compromising cell viability (Figure S1D). This aligns with previous studies reporting broad antiviral activity of BIM‐I not only against influenza A and B viruses [21], but also against HCV [45], SARS‐CoV‐2 [25], porcine reproductive and respiratory syndrome virus [45]. Given its potent antiviral properties, BIM‐I was selected for further investigation as a potential therapeutic candidate against IAV infections. However, significant cytotoxic effects were observed after treatment with 5 to 10 µM BIM‐I (Figure S1D), underscoring the need to improve its cellular tolerability. To address the concentration‐dependent cytotoxicity and hydrophobicity of BIM‐I, which constrain its therapeutic applicability, the compound was encapsulated in NPs to improve its suitability for potential drug delivery applications.

### 2.2 Formulation and Characterization of BIM‐I‐Loaded PLGA NPs

**FIGURE 2:**
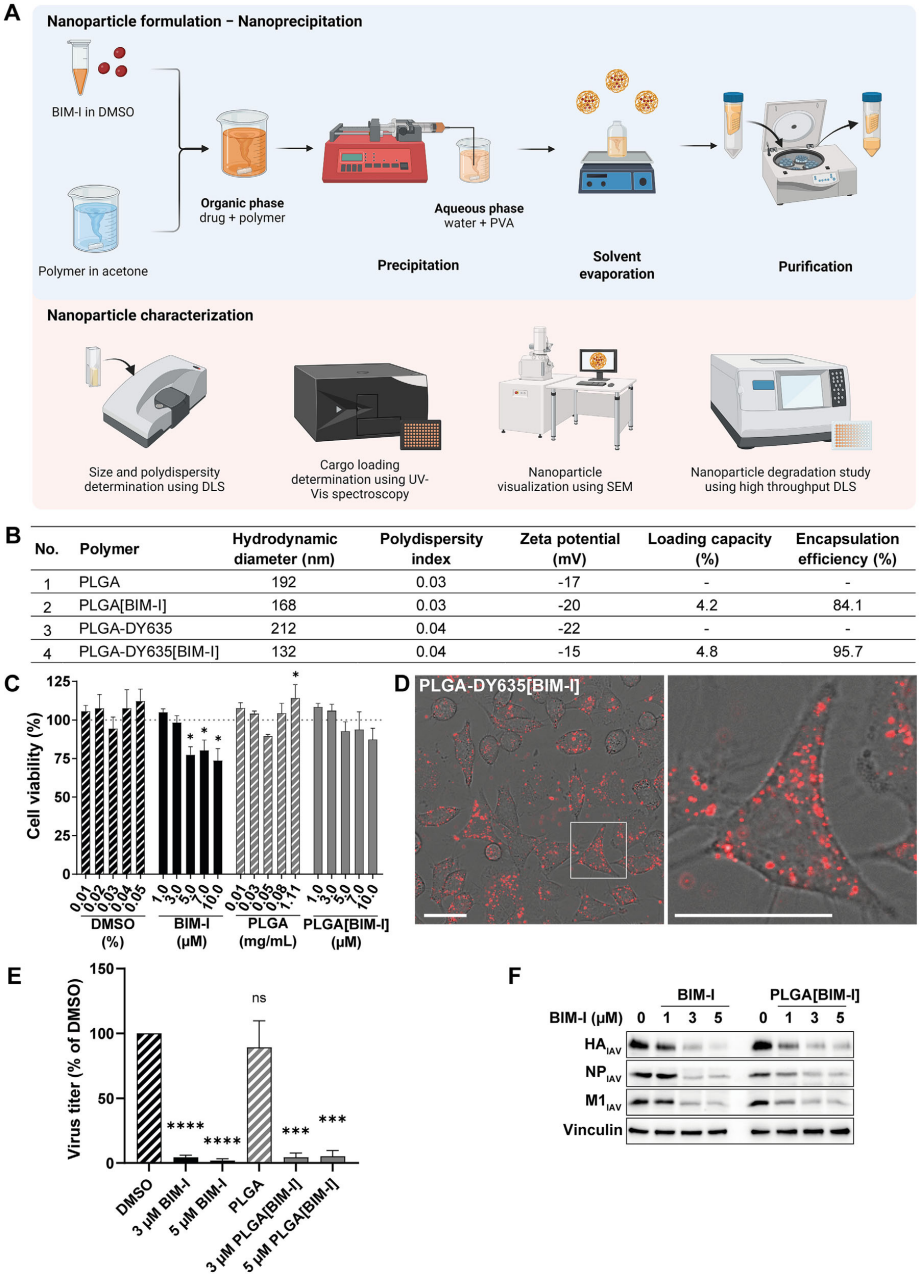
Formulation, characterization, and in vitro efficacy of PLGA[BIM‐I] NPs. A) Workflow of NP formulation and characterization. B) Characteristics of the BIM‐I‐loaded PLGA NPs (PLGA[BIM‐I]). C) Cell viability of A549 cells incubated for 24 h with the indicated concentrations of DMSO, free BIM‐I, blank PLGA NPs, PLGA[BIM‐I] NPs, or with medium alone as a negative control. Blank PLGA NPs correspond to the NP concentrations of PLGA[BIM‐I] NPs. Values were normalized to the medium‐treated control, which was set to 100%. The mean + SD of n=3 independent experiments, each including three technical replicates, is shown. Statistical significance was determined using two‐way ANOVA followed by Dunnett's multiple comparison test. D) Representative images from laser scanning microscopy of A549 cells incubated with 0.1 mg mL^−1^ PLGA‐DY635[BIM‐I] NPs for 2 h (DY635 signal depicted in red; scale bar: 10 µm). E,F) A549 cells were infected with the IAV strain A/PR8 (H1N1) at an MOI of 1 for 30 min. Subsequently, the virus was removed, and the cells were incubated with indicated concentrations of DMSO, free BIM‐I, blank PLGA NPs or PLGA[BIM‐I] NPs for 24 h. Viral titers were determined by plaque assay (E), and the expression of the viral proteins hemagglutinin (HA_IAV_), nucleoprotein (NP_IAV_), and matrix protein 1 (M1_IAV_) were analyzed by Western blot (F). Virus titers were normalized to DMSO, which was set to 100%. The mean + SD of n=3 independent experiments (E,F), each with two biological replicates for the viral titers (E), are shown. Statistical significance was determined using one‐sample *t*‐test relative to 100. *p<0.05, ***p<0.001, ****p<0.0001.

To design BIM‐I‐loaded NP formulations, preparation conditions were adapted from Shkodra et al. [46, 47] and further optimized and refined to meet study‐specific requirements (Figure 2A). Initial NPs were prepared using PLGA as a standard polymer, based on previous reports demonstrating effective BIM‐I encapsulation, efficient PKC inhibition by BIM‐I‐loaded NPs in vitro as well as their hepatic uptake in an in vivo mouse model [46, 47]. For cellular uptake screening, PLGA covalently coupled to the dye DY635 was used to formulate BIM‐I‐loaded NPs, enabling visualization by confocal microscopy. The resulting NPs exhibited sizes (hydrodynamic diameter) between 132 and 212 nm (Figure 2B; Figure S2A–D). The polydispersity indices, reflecting particle size heterogeneity, were low (0.03 to 0.04), indicating uniform size distribution. The negative surface charge (zeta potential) between –15 and –22 mV enhances the colloidal stability of the NPs by promoting electrostatic repulsion, thereby minimizing aggregation and supporting more efficient drug delivery [48]. Encapsulation efficiencies were high, ranging from 84.1 to 95.7%, confirming a robust loading capacity of the formulations (Figure 2B), with consistent drug loading between 4.2 and 4.8%. The physicochemical properties of the NPs were in accordance with previously reported data [46, 47] demonstrating high reproducibility of the NP formulations.

### 2.3 BIM‐I Encapsulation in PLGA NPs Maintains Antiviral Activity while Reducing Cytotoxic Effects

To evaluate possible cytotoxic effects of the NPs, a CellTiter‐Blue Cell Viability Assay was performed. To assess cytotoxicity, A549 cells were incubated with free BIM‐I or BIM‐I‐loaded PLGA NPs as well as DMSO or blank PLGA NPs, serving as the respective negative control, for 24 h. Treatment with the respective concentrations of the blank PLGA NPs had no effect on cell viability (Figure 2C), indicating that the PLGA carrier material does not impair cellular metabolism. This observation aligns with the well‐established biocompatibility and biodegradability of PLGA, which minimizes long‐term accumulation in the body [30]. While treatment with free BIM‐I resulted in dose‐dependent cytotoxicity, reducing cell viability to 73.6% at 10 µM, encapsulation of BIM‐I into PLGA NPs (PLGA[BIM‐I]) mitigated cytotoxic effects, improving cell viability to 87.3% at the corresponding concentration (Figure 2C). This allows the use of BIM‐I at higher concentrations without compromising cellular metabolic activity. As defined by DIN EN ISO 10993‐5, cell viability above 70% is considered non‐cytotoxic. Thus, BIM‐I concentrations up to 10 µM can be used without major cytotoxic effects (Figure 2C), while encapsulated BIM‐I enables the application of even higher concentrations in accordance with the DIN standard. The observed effects of BIM‐I on the cell viability may be attributed to the multifaceted role of PKC in various cellular signaling pathways, including those regulating proliferation, differentiation, migration, survival, and apoptosis [14]. Notably, clinical trials involving PKC inhibitors have reported adverse effects such as nausea, vomiting, fatigue, and diarrhea [49, 50, 51] underscoring the need for alternative delivery strategies to enhance tolerability. Pulmonary drug delivery presents a promising approach for the treatment of respiratory diseases like IAV infections by enabling localized drug administration at the site of infection [52]. This strategy offers the potential to achieve effective therapeutic concentrations while minimizing systemic exposure and associated side effects. Several in vivo studies have already demonstrated the safety and favorable biodistribution of pulmonary delivery via PLGA‐based NPs, further supporting their potential as a targeted and safer alternative to conventional systemic administration [53, 54, 55].

To assess the cellular uptake of NPs, the BIM‐I‐containing, DY635‐labeled NPs (PLGA‐DY635[BIM‐I]) (Figure 2B) were utilized. A549 cells were incubated with 0.1 mg mL^−^1 PLGA‐DY635[BIM‐I] for 2 h, subsequently washed to remove extracellular NPs, and analyzed by confocal laser scanning microscopy. A high number of fluorescently labeled NPs were detected inside the cells, indicating rapid and efficient internalization (Figure 2D).

To examine the antiviral properties of encapsulated BIM‐I, the compound was tested under the same conditions as described above (Section 3.1), using A549 cells. The addition of blank PLGA NPs had no significant effect on viral titers compared to the DMSO control (Figure 2E). In contrast, both free BIM‐I and PLGA‐encapsulated BIM‐I significantly reduced viral titers at concentrations of 3 and 5 µM (Figure 2E), suggesting that BIM‐I is released from the PLGA NPs. Furthermore, treatment with both free and encapsulated BIM‐I significantly decreased the expression of the viral proteins HA_IAV_, nucleoprotein (NP_IAV_) and matrix protein 1 (M1_IAV_), whereas blank PLGA NPs had no effect (Figure 2F). Collectively, these findings demonstrate that PLGA‐encapsulated BIM‐I reduces IAV infection in A549 cells with efficacy comparable to the free inhibitor while exhibiting lower cytotoxicity. The NPs were efficiently taken up and released BIM‐I within the cells at levels sufficient to exert antiviral effects.

### 2.4 Formulation and Characterization of PEGylated and PEtOxylated PLGA NPs Loaded with BIM‐I

To prevent opsonization and to prolong the circulation time of the nanocarriers, “stealth” polymers, most notably PEG, are commonly incorporated into the NP design [56]. Despite its use in approved formulations such as Onivyde, Onpattro and Comirnaty, rising concerns over PEG immunogenicity due to anti‐PEG antibodies have prompted the search for alternatives [57]. Therefore, we explored PEtOx as a stealth alternative for its suitability in BIM‐I delivery due to its comparable physicochemical properties [39].

**FIGURE 3:**
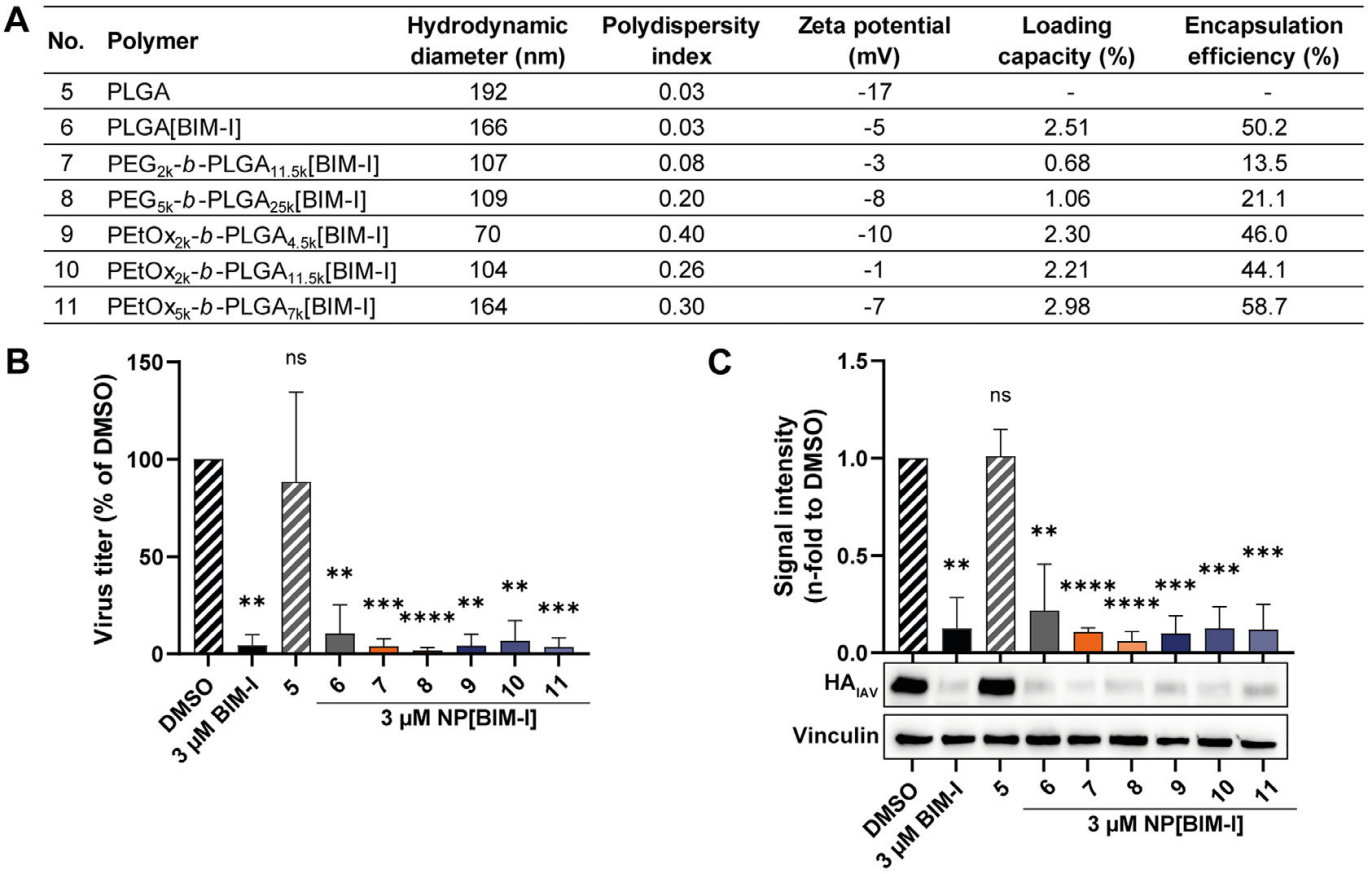
Incorporation of PEG and PEtOx into PLGA[BIM‐I] enhances the inhibitory effect on IAV infection in A549 cells. (A) Characteristics of the BIM‐I‐loaded NPs. (B,C) After infecting A549 cells with the IAV strain A/PR8 (H1N1) at an MOI of 1 for 30 min, the virus was removed, and the cells were incubated with the indicated treatments for 24 h. Viral titers were determined by plaque assay (B). The expression of the viral protein hemagglutinin (HA_IAV_) was analyzed by Western blot and quantified using Fiji ImageJ (C). Virus titers (B) and viral protein expression (C) were normalized to DMSO, which was set to 100% for titers (B) or 1 for protein expression (C). Graphs show the mean + SD of n=3 independent experiments (B,C), each with two biological replicates (B). Statistical significance was determined using one one‐sample *t*‐test relative to 100 (B) or 1 (C). **p<0.01, ***p<0.001, ****p<0.0001.

A series of PEG‐*b*‐PLGA and PEtOx‐*b*‐PLGA copolymers with varying average molar masses were evaluated to assess their influence on the NP characteristics (Figure 3A; Figure S2E–K). The hydrophilic PEG or PEtOx block featured a molar mass of 2 or 5 kDa. The molar mass of the hydrophobic PLGA block was always higher (4.5 to 25 kDa) to favor the formation of nanoparticles instead of micelles or vesicles. The molar masses of the individual blocks is denoted in subscript and was found to be a critical determinant of particle size. Specifically, the incorporation of PEG or PEtOx, along with a reduction in the molar mass of the PLGA segment, resulted in smaller particle sizes, ranging from 70 to 109 nm, except for PEtOx_5k_‐*b*‐PLGA_7k_[BIM‐I], which exhibited a particle size of 164 nm. For this formulation, DLS revealed a bimodal size distribution (Figure S2K), likely due to the high fraction of hydrophilic PEtOx in the block copolymer, which may have prompted the formation of self‐assembled structures, possibly corresponding to micelle‐like structures. Notably, all PEtOx‐containing NP formulations displayed less uniform size distributions, as indicated by their higher polydispersity index values (Figure 3A). Across all formulations, particle sizes ranged from approximately 70 to 190 nm, with NPs solely based on PLGA yielding the largest diameters (Figure 3A; Figure S2E‐K).

To improve the NP purification, ultrafiltration using Amicon filters was introduced as conventional centrifugation was insufficient for sedimenting smaller NPs. This method was uniformly applied to all formulations (Figure 3A, No. 5‐11), including PLGA‐only NPs, to ensure experimental consistency. As ultrafiltration exhibits higher efficiency in removing unencapsulated drug and residual poly(vinyl alcohol) (PVA), it may have contributed to the observed decrease in encapsulation efficiency (84.1% Figure 2B; 50.2% Figure 3A). Formulations based on PEG‐*b*‐PLGA exhibited lower encapsulation efficiencies (13.5 to 21.1%) compared to PEtOx‐*b*‐PLGA NPs (44.1 to 58.7%) (Figure 3A), suggesting a potential advantage of PEtOx in enhancing drug loading capacity.

### 2.5 Incorporation of PEG and PEtOx into PLGA[BIM‐I] Enhances the Inhibitory Effect on IAV Infection in A549 cells

The antiviral efficacy of BIM‐I encapsulated in different variants of PLGA, PEG‐*b*‐PLGA and PEtOx‐*b*‐PLGA was evaluated in A549 cells, as described above (Section 3.1). In comparison to the DMSO control, treatment with blank PLGA NPs revealed no significant impact on viral titers or HA_IAV_ protein expression (Figure 3B, C), indicating that the carrier itself did not interfere with viral replication. Application of 3 µM free BIM‐I led to a significant reduction in viral titers by 95.7%, compared to the DMSO control (Figure 3B). Treatment with 3 µM BIM‐I encapsulated in PLGA NPs also resulted in a strong antiviral effect, albeit to a slightly lesser extent than the free compound, exhibiting an 89.5% reduction in viral load (Figure 3B). This reduced efficacy relative to the free drug may be attributed to a delayed onset of BIM‐I activity, as NP‐mediated delivery requires cellular uptake of NPs followed by intracellular release of the active compound. To enhance the delivery system, BIM‐I was encapsulated in PEGylated PLGA NPs using two different PEG‐*b*‐PLGA copolymers. Treatment with both PEG_2k_‐*b*‐PLGA_11.5k_[BIM‐I] and PEG_5k_‐*b*‐PLGA_25k_[BIM‐I] formulations, each applied at a concentration of 3 µM, led to superior antiviral activity in comparison to PLGA[BIM‐I], reducing viral titers by 96.2% and 98.2%, respectively (Figure 3B). These results indicate that PEGylation might improve cellular uptake or facilitate more efficient release of the encapsulated drug. Further, to investigate the potential of alternative stealth polymers, BIM‐I was encapsulated in PLGA NPs modified with PEtOx chains of varying lengths. All three BIM‐I‐loaded PEtOx‐*b*‐PLGA formulations (PEtOx_2k_‐*b*‐PLGA_4.5k_[BIM‐I], PEtOx_5k_‐*b*‐PLGA_7k_[BIM‐I], and PEtOx_2k_‐*b*‐PLGA_11.5k_[BIM‐I]) significantly suppressed viral replication at a concentration of 3 µM, decreasing viral loads by 95.8%, 93.2% and 96.5%, respectively (Figure 3B). The observed reductions in viral titers across all treatment groups were mirrored by a corresponding decrease in HA_IAV_ protein expression (Figure 3C), confirming that the antiviral effect was associated with impaired viral replication at the protein level as well. These findings underscore the efficiency of PEtOx as an alternative stealth polymer to PEG in drug delivery systems, demonstrating a comparable antiviral effect relative to free BIM‐I.

### 2.6 Formulation and Characterization of PLGA, PEG_2k_‐*b*‐PLGA_11.5k,_ and PEtOx_1.8k_‐*b*‐PLGA_16.8k_ NPs for BIM‐I encapsulation

**FIGURE 4:**
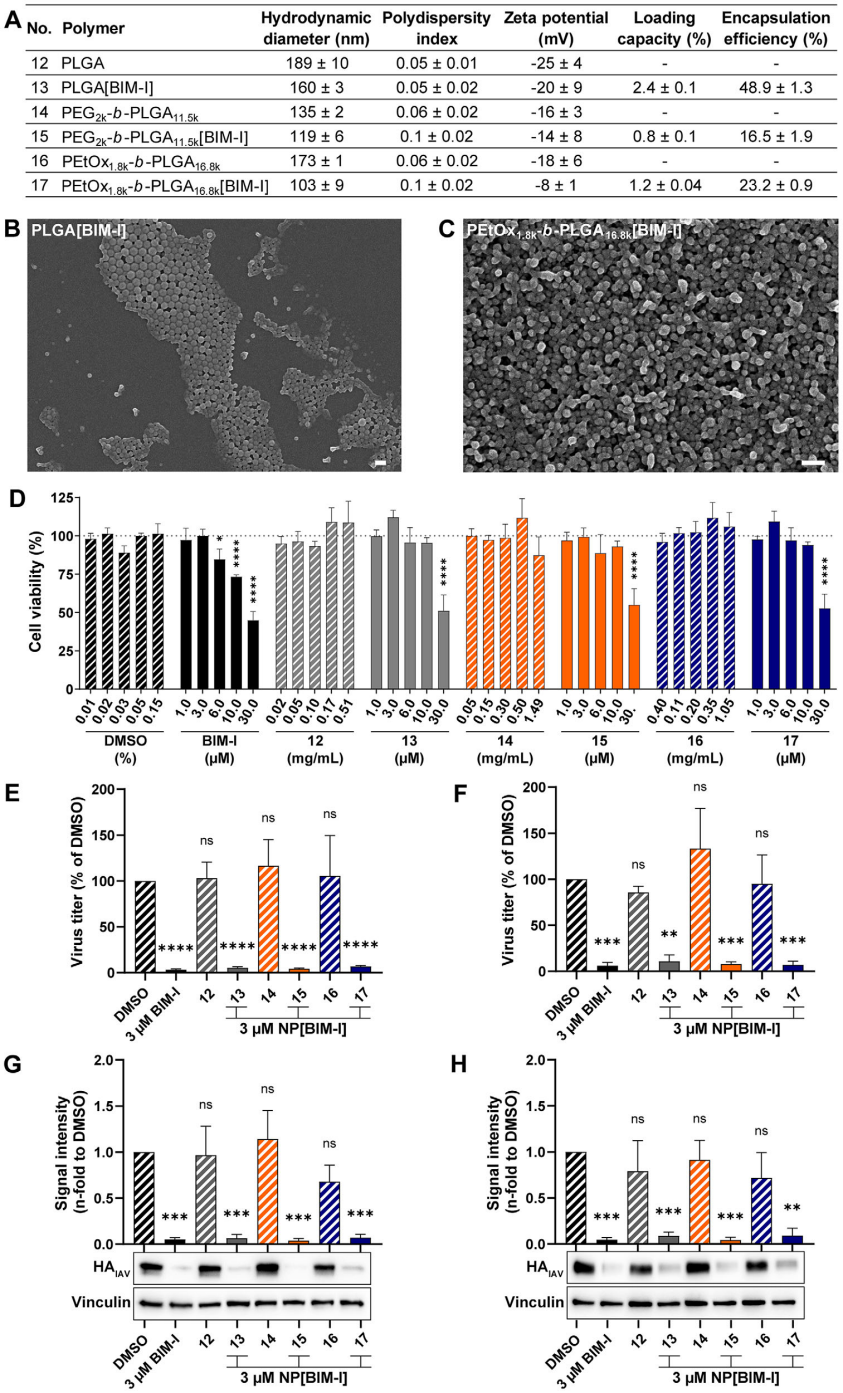
BIM‐I encapsulation by stealth polymer‐containing PLGA NP systems reduces cytotoxic effects while maintaining antiviral activity. A) Characteristics of the BIM‐I‐loaded NPs according to the polymer of choice. The mean ± SD of three independent formulations is shown. B,C) SEM image of BIM‐I‐loaded PLGA (B) and PEtOx_1.8k_‐*b*‐PLGA_16.8k_ NPs (C) (scale bar: 200 nm). D) A549 cells were treated for 24 h with the indicated concentrations of DMSO, free BIM‐I, encapsulated BIM‐I (NP[BIM‐I]), blank NPs or with medium alone as a negative control. Blank NP controls matched the NP concentrations used for BIM‐I‐loaded NPs. Cytotoxicity of the different treatments was analyzed using the CellTiter‐Blue Cell Viability Assay and normalized to the medium‐treated control, which was set to 100%. The data represent the mean + SD of n=3 independent experiments, with three technical replicates. Statistical significance was determined using two‐way ANOVA followed by Dunnett's multiple comparison test. E‐H) Calu‐3 cells (E,G) or MDCK cells (F,H) were infected with the IAV strain A/PR8 (H1N1) at an MOI of 0.1 for 30 min. Subsequently, the virus was removed, and the cells were incubated with 3 µM free or encapsulated BIM‐I or the respective negative control (DMSO or blank NPs) for 24 h. Viral titers were determined by plaque assay (E,F) and the expression of the viral protein hemagglutinin (HA_IAV_) was analyzed by Western blot (G,H). Virus titers and viral protein expression were normalized to DMSO, which was set to 100% (E,F) or 1 (G,H). The mean + SD of n=3 independent experiments (E–H), with two biological replicates for viral titers (E,F), are shown. Statistical significance was determined using one sample t‐test comparing to 100 for viral titers (E,F) or 1 for protein expression (G,H). *p<0.05, **p<0.01, ***p<0.001, ****p<0.0001.

Based on the efficacy of the previous BIM‐I‐loaded NPs (Figure 3), additional formulations were prepared with further optimization. For further investigation, the following three NP systems were selected: 1) PLGA, 2) PEG_2k_‐*b*‐PLGA_11.5k,_ and 3) PEtOx_1.8k_‐*b*‐PLGA_16.8k_. PLGA and PEG_2k_‐*b*‐PLGA_11.5k_ were chosen based on their favorable performance in preliminary assessments (Figure 3). PEtOx_1.8k_‐*b*‐PLGA_16.8k_ was synthesized to closely match the molar mass of PEG_2k_‐*b*‐PLGA_11.5k_ and to extend the hydrophobic PLGA block, aiming to achieve more uniform NP dispersions with a low polydispersity index and without a tendency to assemble into smaller micelles or vesicles. Therefore, NPs were reformulated as described for NPs No. 5‐11 (Figure 3A), confirming the method's reproducibility based on consistent particle characteristics (Figure 4A). The NP sizes ranged from 100 to 190 nm with low polydispersity indices (0.01 to 0.1) (Figure 4A; Figure S2L–Q), now verifying narrow size distribution for all samples. The encapsulation efficiency of BIM‐I varied, with PLGA achieving the highest (48.9%), while lower but acceptable encapsulation was obtained with the other systems (16.5% with PEG_2k_‐*b*‐PLGA_11.5k_[BIM‐I] and 23.2% with PEtOx_1.8k_‐*b*‐PLGA_16.8k_[BIM‐I]) (Figure 4A). Notably, PEtOx‐functionalized NPs demonstrated comparable or even superior encapsulation efficiency relative to the commonly used PEG‐containing formulations (Figures 3A and 4A), highlighting their potential as a novel alternative to PEG. The overall reduced encapsulation efficiency of stealth polymer‐modified NPs may be attributed to the smaller particle size, a phenomenon also observed in previous studies [58, 59, 60]. The decrease in the loading capacity of the PLGA NPs from the previously reported results (Figure 2B) is associated with the improved NP purification by ultrafiltration using Amicon filters.

Morphological characterization of the PLGA[BIM‐I] and PEtOx_1.8k_‐*b*‐PLGA_16.8k_[BIM‐I] NPs by SEM revealed spherical and uniform structures (Figure 4B,C), consistent with the size and low polydispersity index data obtained from DLS measurements (Figure 4A). The introduction of hydrophilic PEG and PEtOx segments increased the overall hydrophilicity of the NPs, which introduced challenges during SEM imaging. In particular, PEG_2k_‐*b*‐PLGA_11.5k_[BIM‐I] NPs exhibited blurred boundaries when deposited on dried mica disks, likely due to their high surface hydration, making them less suitable for SEM analysis.

To evaluate NP degradation under biological conditions, Proteinase K is frequently employed to mimic enzymatic environments encountered in vivo. Proteinase K is a broad‐spectrum serine protease that catalyzes the hydrolysis of peptide bonds, enabling assessment of NP stability and degradation in proteolytic settings [61]. The degradation profiles of the three NP systems were analyzed using a two‐phase decay model, which provided an appropriate representation to describe the observed data. PEG_2k_‐*b*‐PLGA_11.5k_ and PEtOx_1.8k_‐*b*‐PLGA_16.8k_ NPs exhibited a slightly faster degradation rate compared to PLGA NPs (Figure S3A‐C), likely due to the increased hydration of the PEG‐ and PEtOx‐functionalized NPs, which enhances their susceptibility to enzymatic hydrolysis [62, 63, 64]. Additionally, due to their smaller particle sizes and consequently higher surface area‐to‐volume ratios, PEG‐ and PEtOx‐containing NPs are more susceptible to degradation [65]. These results underscore the biodegradable and biocompatible properties of these NP drug delivery systems, making them promising candidates for potential biomedical applications.

### 2.7 BIM‐I Encapsulation by Stealth Polymer‐Containing PLGA NP Systems Reduces the Compounds Cytotoxic Effects in A549 Cells

To evaluate the cytocompatibility of the developed NP systems and the impact of BIM‐I encapsulation on the cell viability, a CellTiter‐Blue Cell Viability Assay was performed in A549 cells after 24 h incubation. To assess the potential cytotoxicity of the nanocarriers themselves, blank NPs were applied at concentrations equivalent to those of the BIM‐I‐loaded formulations. No significant reduction in cell viability was observed for blank PLGA, PEG_2k_‐*b*‐PLGA_11.5k_ and PEtOx_1.8k_‐*b*‐PLGA_16.8k_ NPs up to concentrations of 0.51, 1.49, and 1.05 mg mL^−1^, respectively, indicating that the carrier systems are well tolerated by A549 cells (Figure 4D). These findings align with previous studies, demonstrating the biosafety of PEG‐*b*‐PLGA NPs following intravenous [66, 67] intraperitoneal, [68] oral, [69] nasal [58, 66] and intratracheal [70, 71] administration in vivo. Furthermore, the safety of PEtOx has been extensively evaluated in a recent clinical trial (NCT02579473) involving subcutaneous administration of a PEtOx‐conjugated drug candidate for Parkinson's disease, which achieved sustained drug release, [72] further supporting the polymer's biocompatibility and translational potential. Concentrations of up to 3 µM of free and encapsulated BIM‐I did not display significant cytotoxic effects (Figure 4D). Treatment with 6 or 10 µM free BIM‐I resulted in a significant reduction in cell viability to 84.7% and 73.3%, respectively (Figure 4D), which remains within the acceptable range of non‐cytotoxicity as defined by DIN EN ISO 10993‐5. In contrast, BIM‐I encapsulated into all three PLGA‐based NP formulations mitigated these cytotoxic effects, maintaining cell viability above 93% at the same BIM‐I concentration (Figure 4D). At a higher dose of 30 µM, all BIM‐I‐containing treatments induced significant cytotoxicity. Free BIM‐I reduced cell viability to 45%, while encapsulation of BIM‐I in NPs resulted in higher cell viability levels above 51% (Figure 4D). The enhanced cell viability is likely attributable to a slower and more sustained release of BIM‐I from the NP carriers, thereby reducing peak drug concentrations that are often associated with cytotoxicity. From a clinical perspective, the use of such NP‐based delivery systems could offer substantial advantages by minimizing drug‐related toxicity and enhancing patient compliance through reduced dosing frequency [73].

Together, these findings demonstrate that NP‐mediated delivery of BIM‐I effectively reduces its cytotoxic profile. Furthermore, all tested NP systems exhibit high cytocompatibility, underscoring their suitability as vehicles for drug delivery.

### 2.8 BIM‐I Encapsulation by Stealth Polymer‐Containing PLGA NP Systems Maintains Antiviral Activity in Calu‐3 and MDCK cells

The antiviral activity of BIM‐I, encapsulated in three distinct polymer systems (Figure 4A), was further evaluated in two additional physiologically relevant cell lines infected with the IAV strain A/PR8 (H1N1) and subsequently treated with 3 µM free or encapsulated BIM‐I, or the respective negative controls, as described above (Section 3.1). Accordingly, experiments were conducted in Calu‐3 and MDCK cells (Figure 4E–H). Calu‐3 cells are human bronchial epithelial cells isolated from a lung adenocarcinoma, exhibiting key bronchial features such as tight junction formation, mucin production and cilia expression [74, 75]. MDCK cells are highly susceptible to IAV infection, enabling efficient virus replication, and are therefore commonly used for potency testing of drugs against IAVs [76, 77]. Blank NPs served as controls and did not significantly affect viral titers or HA_IAV_ protein expression in either Calu‐3 or MDCK cells (Figure 4E–H; No. 12, 14, 16), indicating that the polymeric carriers alone do not interfere with IAV replication. In Calu‐3 cells, treatment with free BIM‐I resulted in a 96.9% reduction in viral titers relative to the DMSO control, demonstrating strong antiviral activity (Figure 4E). Similar effects were observed for BIM‐I encapsulated in PLGA (94.9%), PEG_2k_‐*b*‐PLGA_11.5k_ (95.8%), and PEtOx_1.8k_‐*b*‐PLGA_16.8k_ (93.3%) NPs (Figure 4E). All BIM‐I‐containing treatments also significantly reduced the expression of the viral HA_IAV_ protein in Calu‐3 cells (Figure 4G), indicating efficient drug release and comparable antiviral activity across all formulations. In MDCK cells, treatment with each BIM‐I formulation, including the free compound, resulted in significant reductions in viral titers. Free BIM‐I reduced viral titers by 93.9% relative to the DMSO control (Figure 4F). Compared to the PLGA‐only formulation, which suppressed viral load by 89.1%, PEGylated and PEtOxylated formulations achieved slightly improved antiviral efficacy of 92.2% and 92.9%, respectively (Figure 4F). This pattern suggests a potential advantage of stealth polymer modification in enhancing delivery or release kinetics in highly permissive cell types. Consistently, all BIM‐I‐containing treatments significantly diminished HA_IAV_ protein expression in MDCK cells (Figure 4H). Together, these findings demonstrate that BIM‐I retains potent antiviral activity against IAV infection when encapsulated in PLGA‐based NPs, as demonstrated across different cellular systems. Moreover, stealth polymer incorporation may further improve therapeutic performance, particularly in cell types supporting high levels of viral replication, with PEtOx‐functionalized NPs achieving comparable results to the PEGylated formulation and thereby serving as a viable alternative.

### 2.9 PLGA, PEG_2k_‐*b*‐PLGA_11.5k,_ and PEtOx_1.8k_‐*b*‐PLGA_16.8k_ NPs were Efficiently Internalized by Different Cell Lines

**FIGURE 5:**
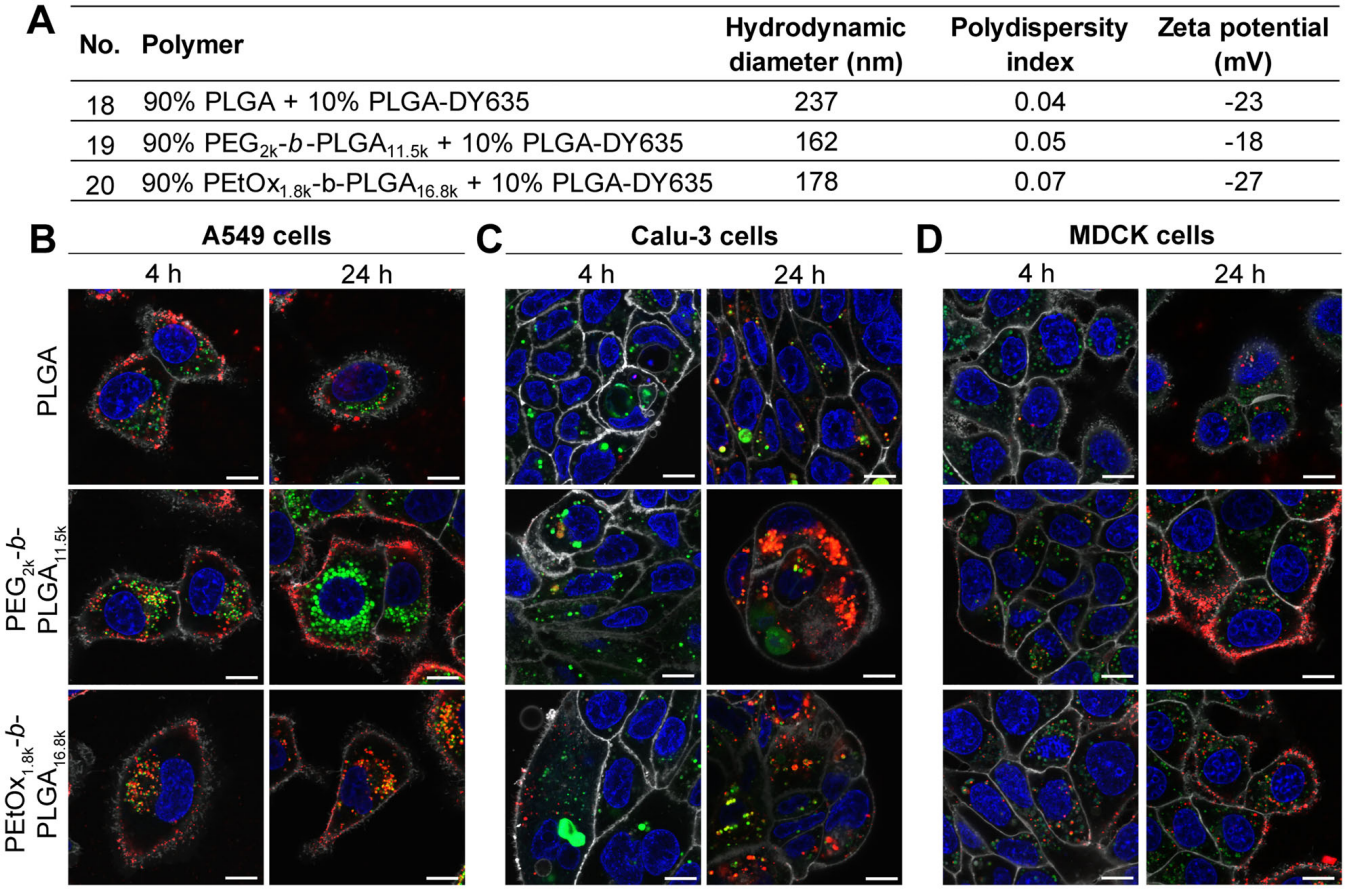
DY635‐labeled PLGA, PEG_2k_‐*b*‐PLGA_11.5k_ and PEtOx_1.8k_‐*b*‐PLGA_16.8k_ NPs were efficiently internalized by different cell lines. A) Properties of DY635‐labeled NPs. B‐D) A549 (B), Calu‐3 (C) or MDCK cells (D) were incubated with 0.1 mg mL^−1^ of respective NPs for 4 or 24 h. Subsequently, cells were washed and stained utilizing different organelle staining. Images were acquired using a confocal laser scanning microscope (red: DY635; green: LysoTrackerGreen DND‐26; blue: Hoechst33342; white: CellMaskOrange Plasma Membrane Stain; scale bar: 10 µm) Representative images of a total of ≥37 cells per condition, obtained from n=1 independent experiments, are shown.

To assess the cellular uptake of the different NP formulations, DY635‐labeled NPs were prepared using PLGA covalently conjugated with the dye DY635. The resulting particles exhibited sizes ranging from approximately 160 to 240 nm, with the largest diameters observed in formulations lacking PEG or PEtOx (Figure 5A; Figure S2R–T). All NPs displayed low polydispersity indices (0.04 to 0.07), indicating uniform size distribution, and exhibited a negative zeta potential (–18 to –27 mV) (Figure 5A). These physicochemical characteristics closely mirrored those of BIM‐I‐loaded NPs (Figure 4A), supporting their use as suitable models for comparative cellular uptake studies. A549, Calu‐3, and MDCK cells were incubated with the labeled NPs for 4 h or 24 h and subsequently washed to remove extracellular NPs. For cellular compartmentalization, nuclei, lysosomes, and plasma membranes were stained using different fluorescent organelle markers and visualized by confocal laser scanning microscopy (Figure 5B‐D). The amount of NPs internalized by cells and localized within lysosomes was quantified using a custom‐built pipeline in the ImageJ‐based visual programming platform JIPipe (Figures S4–S6). In A549 cells, all NP variants revealed efficient and rapid uptake, with strong intracellular signals visible after only 4 h of incubation (Figure 5B; Figure S4A). In contrast, Calu‐3 and MDCK cells displayed slower internalization kinetics, with minimal intracellular fluorescence detectable at the 4 h time point for Calu‐3 cells (Figure 5C, D; Figure S4A). After 24 h incubation, all three DY635‐labeled NP variants (PLGA, PEG_2k_‐*b*‐PLGA_11.5k_ and PEtOx_1.8k_‐*b*‐PLGA_16.8k_) were detected inside all tested cell lines (Figure 5B–D; Figure S4A). The accelerated uptake in A549 cells (Figure 5B) may be attributed to the presence of an activating Kirsten rat sarcoma viral oncogene homologue (KRAS) mutation, which has been shown to enhance macropinocytosis, a nutrient‐scavenging pathway commonly upregulated in cancer cells [78]. Furthermore, the fluorescent NP label DY635 mimics a ligand for organic anion transporting polypeptides (OATPs) OATP1B1 and OATP1B3, which are expressed in hepatocytes and various cancer cells and can thereby serve as a targeting moiety [79, 80]. Notably, both A549 and Calu‐3 cells have been reported to express OATP1B1 and OATP1B3 [81, 82], whereas MDCK cells lack these transporters [83], which may explain the different uptake kinetics across the different cell lines.

Despite the slower internalization in Calu‐3 and MDCK cells (Figure 5C,D; Figure S4A), the BIM‐I‐loaded NPs described above (Figures 3A and 4A) demonstrated comparable antiviral efficacy across all cell types (Figure 3B,C; Figure 4E–H). This functional readout indicates sufficient NP uptake and intracellular drug release within 24 h.

In general, cells incubated with PEG‐ or PEtOx‐containing PLGA NPs exhibited markedly stronger fluorescence signals compared to those treated with unmodified PLGA NPs (Figure 5B–D; Figure S4A), indicating enhanced cellular uptake of stealth polymer‐coated NP formulations. These findings are consistent with previous observations by Kirby et al.et al., who reported enhanced internalization of PEG‐*b*‐PLGA NPs compared to PLGA NPs in colorectal adenocarcinoma (Caco‐2) cells, with NP uptake increasing with the degree of PEGylation [58]. The more efficient internalization may be attributed to the improved colloidal stability provided by PEG and PEtOx, leading to reduced NP aggregation and nonspecific protein adsorption, which, in turn, improves dispersion and NP internalization [56]. Furthermore, the smaller particle sizes of PEGylated (162 nm) and PEtOxylated (178 nm) NPs, relative to unmodified PLGA NPs (237 nm) (Figure 5A), likely contribute to their enhanced internalization. Consistent with our findings, previous studies have demonstrated higher uptake of smaller PLGA NPs across various cell lines as well as in murine lung tissue following systemic administration [84, 85, 86, 87].

The intracellular localization also varied by NP composition and cell line. After 24 h, PEGylated NPs were predominantly localized near the plasma membrane in both A549 and MDCK cells (Figure 5B,D). In contrast, PLGA NPs and PEtOxylated NPs exhibited a broader cytoplasmic distribution. In all tested cell lines, the different NP variants colocalized with lysosomal compartments (Figure S4B). Overall, differences in NP uptake and intracellular distribution were evident across cell types (Figure 5B–D; Figure S4A), potentially reflecting cell line‐specific endocytic mechanisms. This is in line with other literature reports, which have reported different internalization routes and intracellular fates of PLGA NPs across different cell lines [88, 89, 90, 91].

Collectively, these results demonstrate that, while the extent and kinetics of NP uptake vary among cell types and NP formulations, all tested systems are capable of entering target cells and delivering therapeutic cargo. Importantly, NPs functionalized with the stealth polymers PEG or PEtOx exhibited enhanced uptake rates (Figure 4B–D).

## 3 Conclusion

Considering the worldwide health threat posed by IAV infections, the development of effective antiviral therapeutic strategies remains of utmost importance. Targeting host cellular factors that mediate viral replication decreases the likelihood of resistance emergence and therefore represents a promising approach for effective antiviral therapy. In this study, we evaluated the antiviral potential of GRK and PKC inhibitors against IAV infection. Despite reports in literature, the tested GRK inhibitors paroxetine and CMPD101 showed no antiviral efficacy in our system and were therefore excluded from further optimization. In contrast, both PKC inhibitors, Gö6983 and BIM‐I, efficiently reduced IAV infection, with BIM‐I exhibiting potent antiviral activity across different cell lines. To address limitations associated with BIM‐I's poor solubility and cytotoxicity, we encapsulated the compound in various PLGA‐based NP systems. This approach preserved the compound's antiviral efficacy across multiple cellular models while improving its cellular tolerability and translational potential. The observed effects suggest a slower and sustained drug release profile, potentially maintaining therapeutic levels over extended periods. Such sustained release likely contributes to lower toxicity by avoiding peak concentrations, a feature highly advantageous for clinical application. NP‐based drug delivery may reduce dosing frequency and improve tolerability, thereby enhancing patient compliance, particularly in long‐term treatments. Moreover, the potential for pulmonary delivery of PLGA‐based NPs is particularly relevant for respiratory viral infections, including those caused by IAVs, where direct pulmonary delivery could maximize drug efficacy while minimizing systemic side effects [52].

Incorporating stealth polymers into PLGA NPs is essential to prevent opsonization, prolong circulation, and enhance therapeutic potential in clinical applications. While PEG remains the gold‐standard stealth polymer, its increasing association with immunogenicity underscores the urgent need for safer alternatives. In this context, we compared PEG‐*b*‐PLGA and PEtOx‐*b*‐PLGA NPs for BIM‐I encapsulation, demonstrating that PEtOx‐based formulations achieved comparable drug encapsulation efficiency, cytocompatibility, antiviral efficacy and cellular uptake. These results establish PEtOx as a viable stealth alternative and position the novel PEtOx‐*b*‐PLGA NP system as a promising platform for nanocarrier‐based drug delivery. The reproducibility and scalable potential of these formulations further support their suitability for pharmaceutical development.

Collectively, our findings advance the development of stealth nanocarriers for host‐targeted antiviral therapy and highlight encapsulated BIM‐I as a potential candidate for therapeutic intervention against IAV infections. Future studies should include comprehensive biosafety and biodistribution analysis in physiologically relevant models, as well as mechanistic investigations of BIM‐I's mode of action and pharmacokinetics.

## 4 Experimental Section

### 4.1 Materials

Acid‐terminated PLGA (Resomer RG 502 H; 719889) with a ratio of lactic to glycolic units of 50:50 and a molar mass of 7,000 to 17,000 g mol^−1^ was acquired from Evonik. Poly(ethylene glycol) methyl ether‐*block*‐poly(lactic‐*co*‐glycolic) acid polymers PEG_2k_‐*b*‐PLGA_11.5k_ (764760) and PEG_5k_‐*b*‐PLGA_25k_ (799041) were purchased from Sigma‐Aldrich. Poly(2‐ethyl‐2‐oxazolin)‐*block*‐poly(lactic‐*co*‐glycolic) acid (PEtOx_1.8k_‐*b*‐PLGA_16.8k_) was synthesized as described in the Supplementary Information (Figure S7). The synthesis of further PEtOx‐*b*‐PLGA (PEtOx_2k_‐*b*‐PLGA_4.5k_, PEtOx_5k_‐*b*‐PLGA_7k_ and PEtOx_2k_‐*b*‐PLGA_11.5k_) has been discribed by Dirauf et al. [39].

A DY635‐labeled PLGA (PLGA‐DY635) was acquired from SmartDyeLivery. PVA (Mowiol 4‐88, M_w_ ≈ 31 000 g mol^−1^; 81381), proteinase K from *Tritirachium album* (P6556) and 15 mL Amicon Ultra‐4 filters (100,000 g mol^−1^ molecular weight cut‐off (MWCO); UFC9100) were procured from Sigma‐Aldrich. Dimethyl sulfoxide (DMSO, 99.9%+; 022914.K2) and acetone (>99%, extra pure, Acros Organics, 177170010) were purchased from Thermo Fisher Scientific. Paroxetine (Tocris, 2141), CMPD101 (Tocris, 4542), Gö6983 (Tocris, 2285), and BIM‐I (Tocris, 0741) were dissolved in DMSO (≥99.7%, Sigma‐Aldrich, D2650).

#### 4.1.1 NP Formulation

The NPs in this study were prepared using the nanoprecipitation method (Figure 2A). First, 10 mg of the selected polymer (PLGA, PEG‐*b*‐PLGA, or PEtOx‐*b*‐PLGA) was dissolved in 1 mL of acetone and vortexed to ensure complete dissolution. A stock solution of BIM‐I (5 mg mL^−1^) in DMSO was prepared, and 100 µL of this stock was added to the polymer solution to achieve a theoretical drug loading of 5%. The organic phase was thoroughly vortexed and subsequently added dropwise into 10 mL of a 0.3% (w/v) aqueous PVA solution using a syringe pump (World Precision Instruments, Aladdin AL1000‐220) at a flow rate of 2 mL min^−1^. This step was performed under continuous stirring at 800 rpm and at room temperature (RT). Blank NPs were also prepared as controls by replacing the BIM‐I stock solution with an equivalent volume of pure DMSO. For larger scale production, the procedure was repeated, and the resulting yields were pooled to obtain the required volume for applications. Dye‐labeled NPs for cellular uptake studies were prepared using the same procedure with slight modifications: 9 mg of the selected polymer was mixed with 1 mg of PLGA‐DY635, maintaining a 9:1 ratio. No additional cargo was incorporated into these dye‐labeled NPs. The NPs were left stirring overnight to evaporate the acetone.

#### 4.1.2 NP Purification

Centrifugation was performed to purify the NPs by removing excess surfactant, DMSO and non‐encapsulated drug. Initially, for the first batch of formulations, the NPs were transferred to 15 mL Falcon tubes and centrifuged using a swing‐bucket rotor at a fixed speed of 2880 × g for 30 min at 20 °C (Eppendorf, Centrifuge 5804 R). After centrifugation, the supernatant was discarded, and the NP dispersions were washed three times with Milli‐Q water until the supernatant was clear. Finally, the dispersions were resuspended in 1 mL of Milli‐Q water. As the particle size decreased in case of PEG‐ and PEtOx‐*b*‐PLGA, conventional centrifugation proved unsuitable. Consequently, ultrafiltration was adopted. For this method, the NP dispersions were transferred into 15 mL Amicon filters (100 000 g mol^−1^ MWCO) and centrifuged at 4500 × g for 30 min. Following centrifugation, the supernatant was discarded, and the NPs were washed five times with Milli‐Q water until the supernatant was clear, before being resuspended in 1 mL of Milli‐Q water. All samples were stored at 4 °C overnight to ensure complete redispersion before usage and characterization.

#### 4.1.3 NP Characterization

The particle size distribution (hydrodynamic diameter and polydispersity index) and surface charge (zeta potential) of the NPs were characterized using dynamic light scattering (DLS) and electrophoretic light scattering (ELS) with a Zetasizer Ultra (Malvern Panalytical). Measurements employed a laser with a wavelength of 633 nm. Polystyrene UV cuvettes (Brand, 759210) and capillary cuvettes made of polycarbonate (Malvern Panalytical, DTS1070) were used for determining hydrodynamic diameter, polydispersity index, and zeta potential, respectively. The measurements were conducted at a back‐scattering angle of 174.7° and a temperature of 25 °C, incorporating a fluorescence filter. The reported hydrodynamic diameter is calculated based on the intensity‐weighted particle size distribution and reported as a z‐average. NPs were diluted 1:100 in Milli‐Q water, and measurements for hydrodynamic diameter and polydispersity index were repeated five times with 15 runs per measurement, each lasting 1.68 s. Zeta potential was determined from three measurements.

To investigate the degradation behavior of the delivery systems, the NPs were incubated in a 1 mg mL^−1^ proteinase K (PK) solution (in Milli‐Q water) at a mass ratio of NP to PK of 1:5, and the normalized intensity (counts s^−1^) are reported by a high‐throughput DLS (HT‐DLS) DynaPro Plate Reader II (Wyatt Technology) over a period of 24 h. The values represent background‐corrected photon count rates normalized to integration time and instrument parameters, as calculated by the instrument software (DYNAMICS, Wyatt).

To evaluate the encapsulation efficiency and loading capacity of BIM‐I in the NPs, UV–Vis spectroscopy was performed using the Infinite M200 Pro Plate Reader (Tecan Group). For each formulation, 200 µL aliquots were lyophilized, and the dried NPs were dissolved in DMSO in volumes matching the lyophilized aliquots. After stirring for 15 min, UV absorbance measurements were taken at 462 nm using undiluted and diluted solutions (1:2, 1:4). A 100 µL sample from each solution was analyzed in a Quartz 96‐well plate (Hellma, 730‐009‐44) with a 3 × 3 multiple‐read configuration and a 2,000 µm well border. Standard curves for BIM‐I were prepared under the same conditions.

The loading capacity (%) and encapsulation efficiency (%) were calculated using the following formulas:

(1) $$loading\;capacity=\frac{mass\;of\;drug\;recovered}{mass\;of\;total\;NPs\;recovered}\times 100\%$$

(2) $$encapsulation\;efficiency=\frac{loading\;capacity_{calculated}}{loading\;capacity_{theoretical}}\times 100\%$$

#### 4.1.4 Scanning Electron Microscopy (SEM)

The NP imaging was conducted using a Sigma VP field emission scanning electron microscope (Carl Zeiss Microscopy GmbH) equipped with an InLens detector and operated at an accelerating voltage of 4 kV. For the sample preparation, 10 µL of the NP dispersion (at a concentration of 1 mg mL^−1^) was drop‐casted onto a mica substrate, air‐dried, and subsequently coated with a thin 4 nm platinum layer using sputter coating (Safematic, CCU‐010 HV) and then measured.

#### 4.1.5 Cell Culture and Viruses

Adenocarcinomic human alveolar basal epithelial (A549) cells, human lung adenocarcinoma cell line (Calu‐3) cells and Madin‐Darby Canine Kidney (MDCK) cells, originally purchased from ATCC, were cultivated in cell culture media (Dulbecco's Modified Eagle's Medium (DMEM; Sigma‐Aldrich, D6429); 10% fetal calf serum (FCS; Sigma‐Aldrich, F7524); 1% penicillin and streptomycin mixture (P/S; 100 U mL^−1^/0.1 mg mL^−1^, Sigma‐Aldrich, P0781)) at 37 °C with 5% CO_2_. The influenza virus strain A/Puerto Rico/8/1934 (H1N1, kind gift from Stephan Ludwig, Muenster, Germany) was used in this study.

#### 4.1.6 Viral Infection

A549 (0.3×10^6^ cells/well), Calu‐3 (1×10^6^ cells/well), or MDCK (0.35×10^6^ cells/well) cells were seeded into 12‐well plates. After 24 h, cells were washed with phosphate buffered saline (PBS; Anprotec, AC‐BS‐0002) and infected with IAV using the indicated multiplicity of infection (MOI) in PBS_INF_ (PBS, 0.2% bovine serum albumin (BSA; Roth, 9401), 1 mM MgCl_2_, 0.9 mM CaCl_2_) or PBS_INF_ as a negative control at 37 °C and 5% CO_2_ for 30 min. PBS_INF_ was removed, and the cells were incubated in medium_INF_ (DMEM, 0.2% BSA, 1 mM MgCl_2_, 0.9 mM CaCl_2_, 0.17 µg mL^−1^ TPCK trypsin (Sigma‐Aldrich, T1426)) supplemented with the indicated substance at 37 °C and 5% CO_2_ for 24 h. Afterward, supernatants were collected for plaque assay and cells were subjected to Western blot analysis.

#### 4.1.7 Plaque Assay

To analyze the viral titers, 2×10^6^ MDCK cells were seeded into each well of 6‐well plates. After 24 h, cells were washed with PBS and infected with serial dilutions of each sample in PBS_INF_ supplemented with P/S (100 U mL^−1^/0.1 mg mL^−1^, Anprotec, AC‐AS‐0004) at 37 °C and 5% CO_2_ for 30 min. After aspiration, 2 mL soft agar (Minimum essential medium (MEM; Gibco, 21430020) supplemented with 0.2% BSA, 0.01% DEAE Dextran (Sigma‐Aldrich, D9885), 0.2% NaHCO_3_ (Biozym Scientific, BE17‐613E), 100 U mL^−1^/0.1 mg mL^−1^ P/S (Anprotec, AC‐AS‐0004), 0.25 µg mL^−1^ TPCK trypsin (Sigma‐Aldrich, T1426) and 0.9% agar (Oxoid, Thermo Fisher Scientific, LP0028B)) were added, and the plates were incubated at 37 °C and 5% CO_2_ for three days. Plaque‐forming units were determined using neutral red staining (Sigma‐Aldrich, N4638). The DMSO solvent‐treated control for each experiment was defined as 100% and the viral titers were normalized to the DMSO solvent‐treated control.

#### 4.1.8 Western Blot Analysis

Cells were washed twice with PBS and subsequently lysed with Triton lysis buffer (20 mM Tris‐HCl, pH 7.4, 137 mM NaCl, 10% glycerol, 1% Triton X‐100, 2 mM EDTA, 50 mM sodium glycerophosphate, 20 mM sodium pyrophosphate) supplemented with proteinase inhibitors (0.2 mM Pefabloc (Sigma–Aldrich, 76307), 5 µg mL^−1^ aprotinin (Carl Roth, A162), 5 µg mL^−1^ leupeptin (Sigma–Aldrich, L5793), 1 mM sodium vanadate (Sigma‐Aldrich, S6508) and 5 mM benzamidine (Sigma‐Aldrich, 434760)) for 30 min at 4 °C. After centrifugation (14 000 rpm, 15 min, 4 °C), the protein concentration of the cleared lysates was measured using Protein Assay Dye Reagent Concentrate (BioRad, 5000006). The lysates were heated (5 min, 95 °C) with 1:5 6x sample loading buffer (375 mM Tris, pH 6.8, 12% SDS, 30% glycerol, 500 mM DTT, 0.01% bromophenol blue), and equal amounts of protein were subjected to SDS‐PAGE. After transfer onto nitrocellulose membranes, total protein was detected using specific antibodies (rabbit anti‐IAV H1N1 hemagglutinin, GeneTex, GTX127357; mouse anti‐IAV nucleoprotein, BioRad, MCA400; mouse anti‐IAV matrix protein 1, BioRad, MCA401; rabbit anti‐vinculin, Cell Signaling, 13901; goat anti‐rabbit, SeraCare, 5220‐0336; goat anti‐mouse, SeraCare, 5220‐0341). The protein levels were quantified using Fiji ImageJ (version ImageJ 1.53t) and normalized to vinculin loading control and DMSO solvent‐treated control.

#### 4.1.9 Cell Viability Assay

A549 cells (6×10^4^ cells/well) were seeded into black 96‐well plates with clear bottoms (Greiner, 655090). The next day, the cells were incubated in cell culture media (DMEM, 10% FCS, 1% P/S) containing the indicated substances in different concentrations at 37 °C and 5% CO_2_ for 24 h. Afterward, the supernatant was removed, and cell culture media supplemented with CellTiter‐Blue Cell Viability Assay reagent (Promega, G8081) at a ratio of 1:6 was added and incubated at 37 °C and 5% CO_2_ for 1.5 h. The fluorescence (excitation 540 nm, emission 610 nm, gain 90, bottom reading) was measured using a TECAN Infinite 200 plate reader (Tecan Group Ltd.). After subtraction of the blank (wells containing only CellTiter‐Blue solution without cells), all samples were normalized by setting the untreated control to 100%.

#### 4.1.10 Confocal Microscopy

A549 (0.3×10^5^ cells/well), Calu‐3 (1×10^5^ cells/well), or MDCK (0.25×10^5^ cells/well) cells were seeded on chambered coverslips (ibidi GmbH, 80826) in cell culture media (DMEM, 10% FCS, 1% P/S) and incubated overnight at 37 °C and 5% CO_2_. Cells were then incubated with 0.1 mg mL^−1^ DY635‐labeled NPs in medium_INF_ (DMEM, 0.2% BSA, 1 mM MgCl_2_, 0.9 mM CaCl_2_, 0.17 µg mL^−1^ TPCK trypsin) for the indicated time points. Subsequently, cells were washed three times with medium_INF_ to remove extracellular NPs. For cellular staining, the cells were incubated with medium_INF_ containing 5 µg mL^−1^ Hoechst 33342 (Thermo Fischer Scientific, H3570), 50 nM LysoTrackerGreen DND‐26 (Thermo Fischer Scientific, L7526), and 2.5 µg mL^−1^ CellMaskOrange Plasma Membrane Stain (Thermo Fisher Scientific, C10045) for 20 min. Imaging was performed utilizing an inverted laser scanning confocal microscope (DMi8 TCS SP8, Leica microsystems) operated with the Leica Application Suite X (version 3.5.5.19976). Images were acquired with a 63x oil immersion objective, with zoom factor 3, line average 3, and 400 Hz in a 2048 × 2048 pixel format. The Hoechst, Lysotracker, CellMask and DY635 channels were sequentially excited at 405, 488, 561, and 633 nm, respectively and emission was collected in the spectral intervals of 410 to 500 nm with Leica hybrid detector (HyD), 500 to 550 nm with HyD, 570 to 620 nm with photomultiplier tubes (PMT) and 640 to 656 nm with HyD, respectively. Brightfield images were collected with a separate PMT. Images were later processed in Fiji ImageJ (version ImageJ 1.53t) to adjust and set identical contrast values over the entire collection of images for all polymers and time points.

#### 4.1.11 Image Analysis

Confocal microscopy images were acquired as described in the section above, with a zoom factor of 1 and containing five channels (fluorescently labeled cell membranes, nuclei, lysosomes, NPs, and a brightfield image). Each image contained approximately 16 to 180 cells, and at least two images of each condition, obtained from n = 1 independent experiments, were analyzed. Image analysis was performed using JIPipe [92], an ImageJ‐based graphical programming language [93, 94]. The JIPipe‐based image quantification workflow is outlined in Figure S5A, with representative examples of the segmented fluorescence‐labeled components shown in Figure S5B–E. The detailed nodes structure of the JIPipe workflow is presented in Figure S6. Regions of interest (ROIs) corresponding to the cells and NPs were identified and evaluated for spatial overlap to quantify the NP uptake. Positive overlap was defined as a minimum of 70% overlap between the NP ROI and the cell ROI. Similarly, lysosomes overlapping with the cells were identified using the same 70% overlap criterion. Colocalization between NPs and lysosomes was further assessed using a 20% overlap threshold. The quantitative outputs from the JIPipe analysis were exported as CSV files, including the number, areas, and overlapping areas of cells, lysosomes, and NPs.

#### 4.1.12 Bioluminescence Resonance Energy Transfer (BRET) Assay

The β‐arrestin2 recruitment to the β2 adrenergic receptor (β2AR) was performed as described by Drube et al. [44]. Briefly, following the Effectene transfection reagent manual (Qiagen, 301427), A549 cells were transfected with 0.5 µg β2AR (C‐terminally tagged with a NanoLuciferase) and 1 µg β‐arrestin2 (N‐terminally fused with a Halo‐Tag) in 21 cm^2^ dishes. After 24 h, 0.4×10^5^ cells/well were seeded into a poly‐d‐lysine‐coated white 96‐well plate (Brand, 781965) in the presence of Halo‐ligand (Promega, G980A) at a ratio of 1:2000. Technical replicates were seeded in triplicate, and a mock labeling condition (without Halo‐ligand) was included for each transfection. After overnight incubation, cells were washed twice with measuring buffer (MB; 140 mM NaCl, 10 mM HEPES, 5.4 mM KCl, 2 mM CaCl_2_, 1 mM MgCl_2_, pH 7.3). Cells were then incubated with indicated concentrations of GRK inhibitor paroxetine or CMDP101 in MB for 30 min. Subsequently, the NanoLuciferase substrate furimazine (Promega, N157B) was added at a dilution of 1:3,500 in MB. Measurements were conducted utilizing a Synergy Neo2 plate reader (Biotek) with Gen5 software (version 2.09), employing a custom‐made filter (excitation: 541 to 550 nm, emission: 560 to 595 nm, fluorescence filter: 620/15 nm). After recording the baseline for 3 min, 1 µM isoprenaline (Iso) was added, and measurements were continued for 5 min. The mock labeling condition was subtracted from the initial BRET ratio to correct for labeling efficiency. For Halo correction, the post‐ligand stimulation values were divided by the corresponding baseline values. These corrected BRET changes were divided by the vehicle control mean. The final dynamic Δ net BRET change was obtained by normalization to the respective untreated control. Calculations were performed utilizing Excel 16.0. Concentration‐response curves were generated utilizing GraphPad Prism 7.05.

#### 4.1.13 Statistical Analysis

Statistical analysis was conducted using GraphPad Prism 10.4.2. Virus titers were normalized to the DMSO solvent control, which was set to 100%. Data are presented as the mean + standard deviation (SD) of three independent experiments, each comprising two biological replicates. Statistical significance was assessed using a one‐sample t‐test relative to a theoretical mean of 100. Viral protein expression levels were normalized to the DMSO solvent control, defined as 1. Data are presented as the mean + SD of three independent experiments, and statistical significance was determined using a one‐sample t‐test relative to 1. For the cytotoxicity assay, cell viability values obtained from different treatments were normalized to the medium‐treated control, which was set to 100%. Data are presented as the mean + SD of three independent experiments, each including three technical replicates. Statistical significance was evaluated using a two‐way ANOVA followed by Dunnett's multiple comparison test. For Figure S1A‐D, a one‐sample t‐test against a theoretical mean of 100 was performed.

## Funding

German Research Foundation, CRC 1278, project number 316213987, Deutsche Forschungsgemeinschaft, SFB 1278, project number 316213987.

## Conflict of Interest

The authors declare no conflict of interest.

## Supporting information




**Supporting File**: adhm70520‐sup‐0001‐SuppMat.docx.

## Data Availability

The data that support the findings of this study are available from the corresponding author upon reasonable request.
