# Supplementary material for: Evaluating the Antiviral Efficacy of Encapsulated PKC Inhibitor BIM‐I against influenza A Virus Infection
Source: Adv Healthc Mater. 2025 Nov 22;15(8):e04060. doi: 10.1002/adhm.202504060 (PMC12927540; doi:10.1002/adhm.202504060)
Supplement: Supplementary file 1 — Supporting File: adhm70520‐sup‐0001‐SuppMat.docx. [file ADHM-15-0-s001.docx]

Supporting Information

Evaluating the antiviral efficacy of encapsulated PKC inhibitor BIM-I against influenza A virus infection

Laura Klement, Jana Ismail, Josefine Schroeder, Amod Godbole, Johanna Schreiber, Christine Weber, Zoltan Cseresnyes, Marc T. Figge, Bettina Löffler, Ulrich S. Schubert, Stephanie Schubert, Christina Ehrhardt, Carsten Hoffmann*


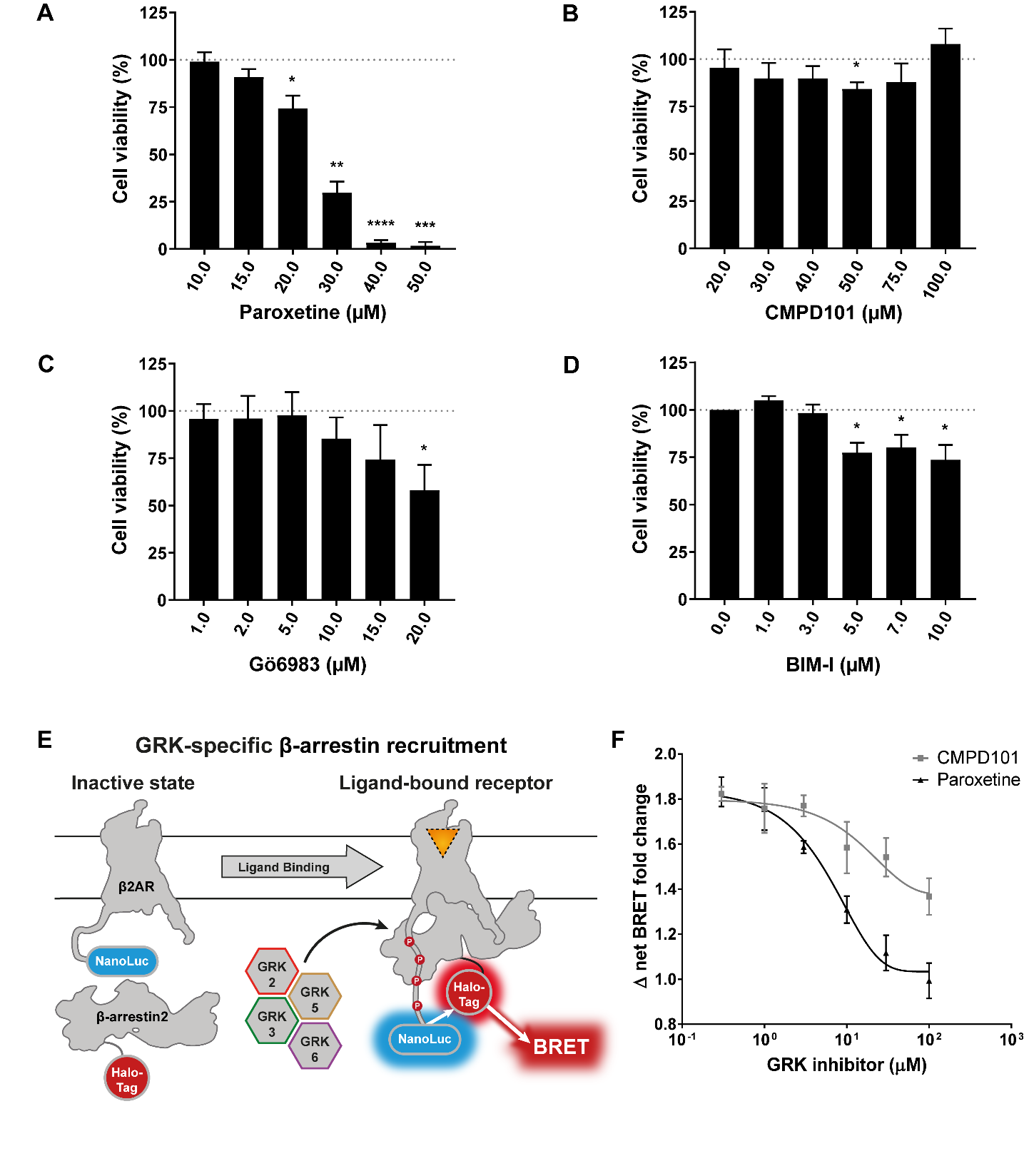


**Figure S1**: A-D) Cytotoxicity of different kinase inhibitors. A549 cells were treated for 24 h with the indicated concentrations of paroxetine (A), CMPD101 (B), Gö6983 (C), BIM-I (D) or with medium alone as a negative control. Cytotoxicity of each inhibitor was assessed using the CellTiter-Blue Cell Viability Assay and normalized to the medium-treated control, which was set to 100%. The data represent the mean + SD of n=3 independent experiments, each performed in triplicate. Statistical significance was determined using one sample t-test relative to 100. *p<0.05, **p<0.01, ***p<0.001, ****p<0.0001. D) shows a subset of the data presented in Fig. 2C. E,F) Efficacy of GRK inhibitors in A549 cells tested using Bioluminescence Resonance Energy Transfer (BRET) assay. E) Schematic representation of NanoBRET-based assay to measure β-arrestin2-NanoLuciferase (NanoLuc) recruitment to β2 adrenergic receptor (β2AR)-Halo-Tag (adapted from Drube *et al.*).^[1]^ Upon stimulation of the β2AR with its ligand isoprenaline (Iso), GRK-mediated phosphorylation promotes β-arrestin2 recruitment to the receptor, enabling energy transfer from the donor NanoLuc to the acceptor Halo-Tag, resulting in a measurable BRET signal. F) Inhibition of GRKs by paroxetine and CMPD101 reduces BRET fold change by impairing receptor phosphorylation and subsequent β-arrestin2 recruitment. A549 cells overexpressing β-arrestin2-NanoLuc and β2AR-Halo-Tag were pre-incubated with the indicated GRK inhibitor concentrations for 30 min and subsequently stimulated with 1 µM Iso. Data are presented as mean ± SEM of n=3 independent experiments, each including three technical replicates.


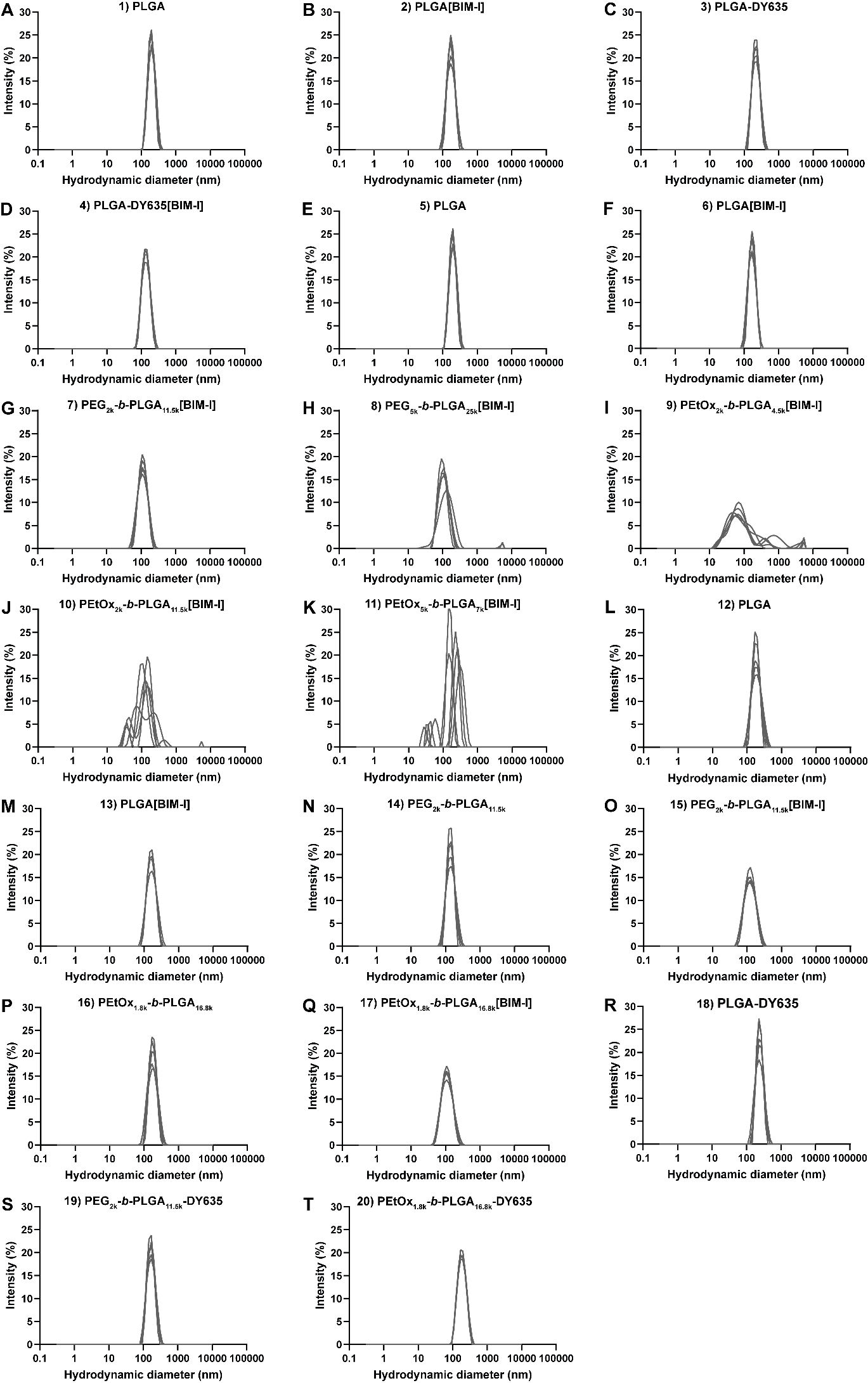


**Figure S2**: Size distribution by intensity of all NP formulations. Samples were diluted 1:100 in Milli-Q water and subsequently analyzed by dynamic light scattering (DLS) using a 633 nm laser wavelength and a back-scattering angle of 174.7°. Each formulation was measured in five replicates. All formulations were prepared as a single experimental replicate (n=1; A-K, R-T), except for those shown in panel L-Q, which were prepared in experimental triplicate (n=3). One representative sample from these triplicates is displayed (L-Q). The polydispersity index and z-average hydrodynamic diameters are reported in Figure 2 (A-D), 3 (E-K), 4 (L-Q) and 5 (R-T).


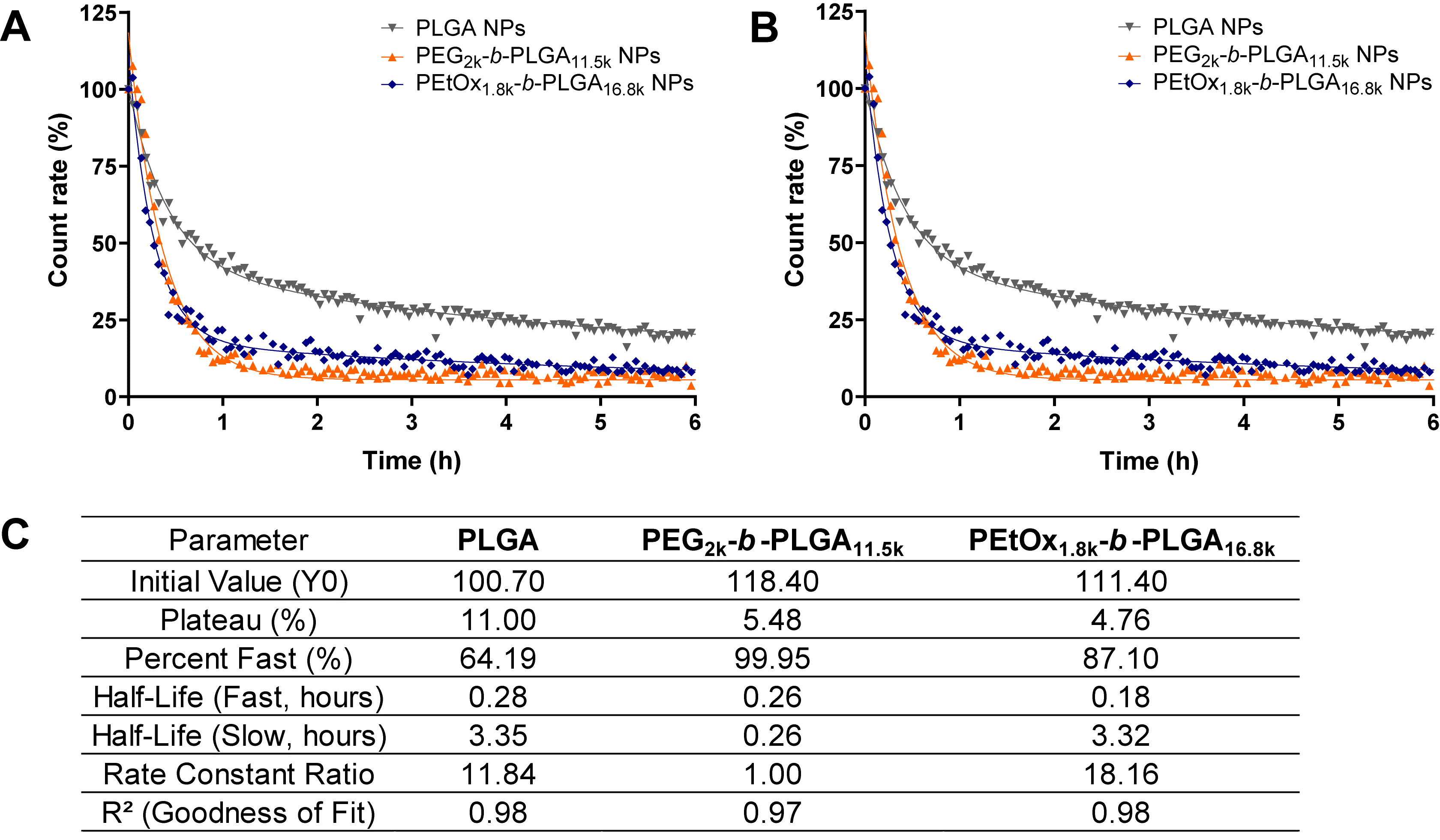


**Figure S3**: Enzymatic degradation of NPs mixed at a 1:5 mass ratio with PK was monitored by determining the count rate via HT-DLS over 6 h (A) and 24 h (B). A,B) Summary of two-phase decay parameters for the three NP systems. C) Results of nonlinear regression analysis of NP decay.


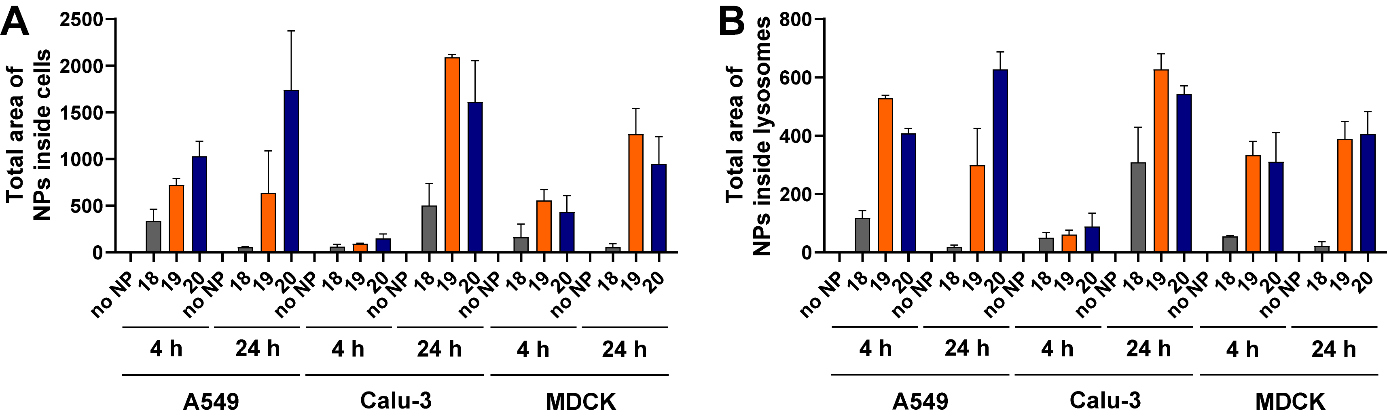


**Figure S4**: NP uptake into cells and lysosomes following incubation with DY-635-labeled NPs. (A,B) A549, Calu-3 and MDCK cells were incubated with 0.1 mg mL^-1^ of the respective NPs (Figure 5A) or medium alone as a negative control (no NP) for 4 h or 24 h. Images were acquired using a confocal laser scanning microscope. Each image contained approximately 16 to 188 cells, and at least two images per condition, obtained from n=1 independent experiment, were analyzed. Image analysis was performed using a custom-built pipeline in the ImageJ-based visual programming platform JIPipe. Regions of interest (ROIs) corresponding to cells, NPs, nuclei and lysosomes were identified and evaluated for spatial overlap to quantify NP uptake. The area of overlap between NPs and cells (A) or between NPs and lysosomes (B) is presented.


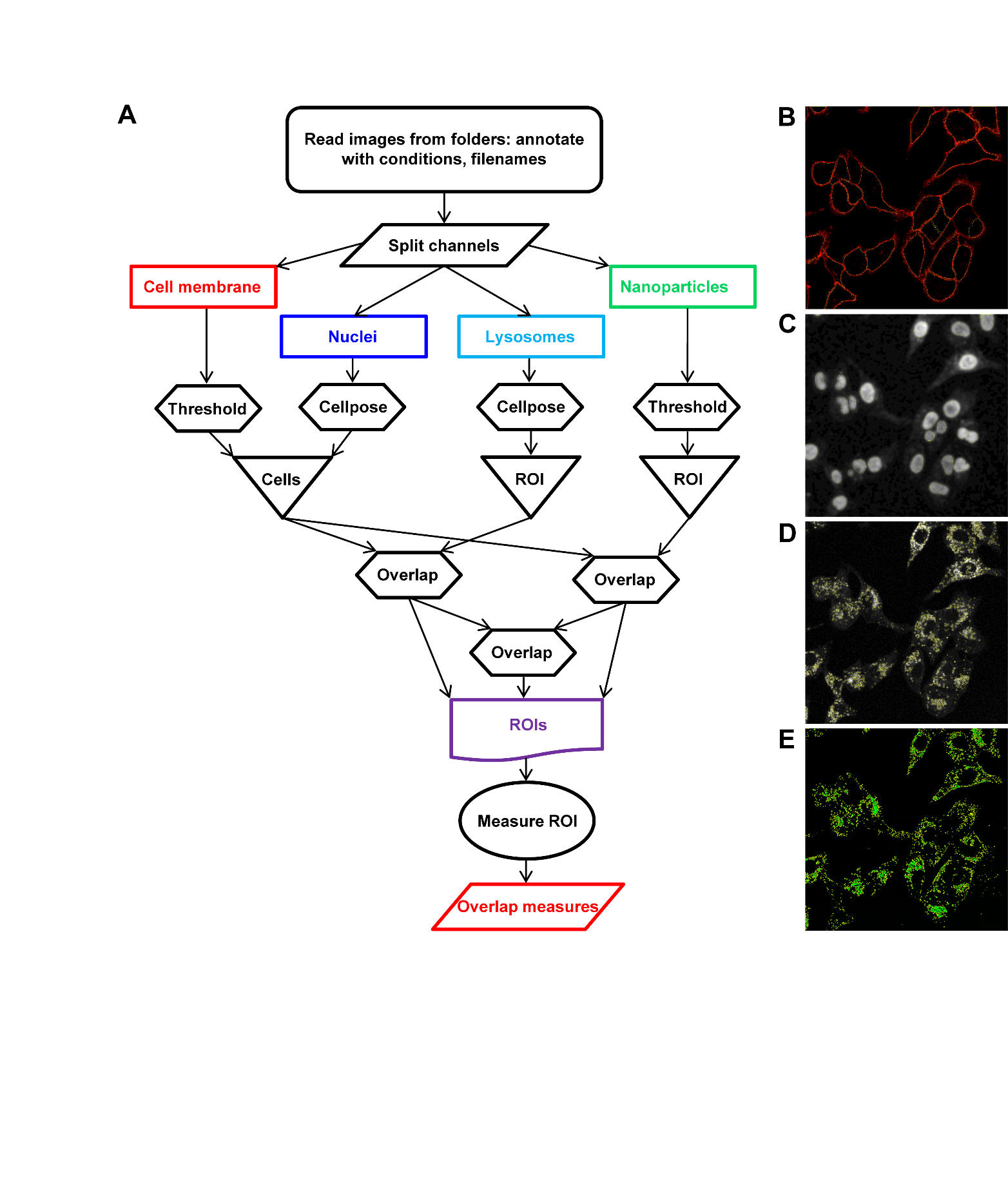


**Figure S5**: The JIPipe-based image quantification workflow, showing the quantification steps applied to the images using a custom-built pipeline in the ImageJ-based^[2]^ visual programming language JIPipe.^[3, 4]^ A) The images were provided in a TIF format, read and annotated (top rounded rectangle). The 5-channel images were split into individual frames, corresponding to images of the cell membrane (red rectangle), the nuclei (blue rectangle), the lysosome image (cyan rectangle), the NP channel (green rectangle), and the brightfield (not shown). The cell membrane and the NP channels were segmented using traditional image analysis techniques based on automated thresholding (cell membrane and NP images in 5-channel recordings). The nuclei and the lysosomes were segmented using Cellpose,^[5]^ applying the pre-trained model “Cytoplasm3” with a radius of 70 pixels or 10 pixels, respectively (black hexagons). The segmented cells were received by using the binarized membrane label channel applying a seeded watershed transformation based on the segmented nuclei channel (black inverted triangle). The lysosomes and the NPs were represented by their regions of interest (ROI, black inverted triangles). The overlap between the lysosomes, the NPs, and the cells was calculated and utilized as a measure of NP uptake into the cells and the lysosomes. B-E) Example images of the segmentation process by the JIPipe workflow. The segmented cells (B), nuclei (C), lysosomes (D) and NPs (E) from A549 cells incubated with NP No. 20 (Figure 5A) for 24 h are shown.


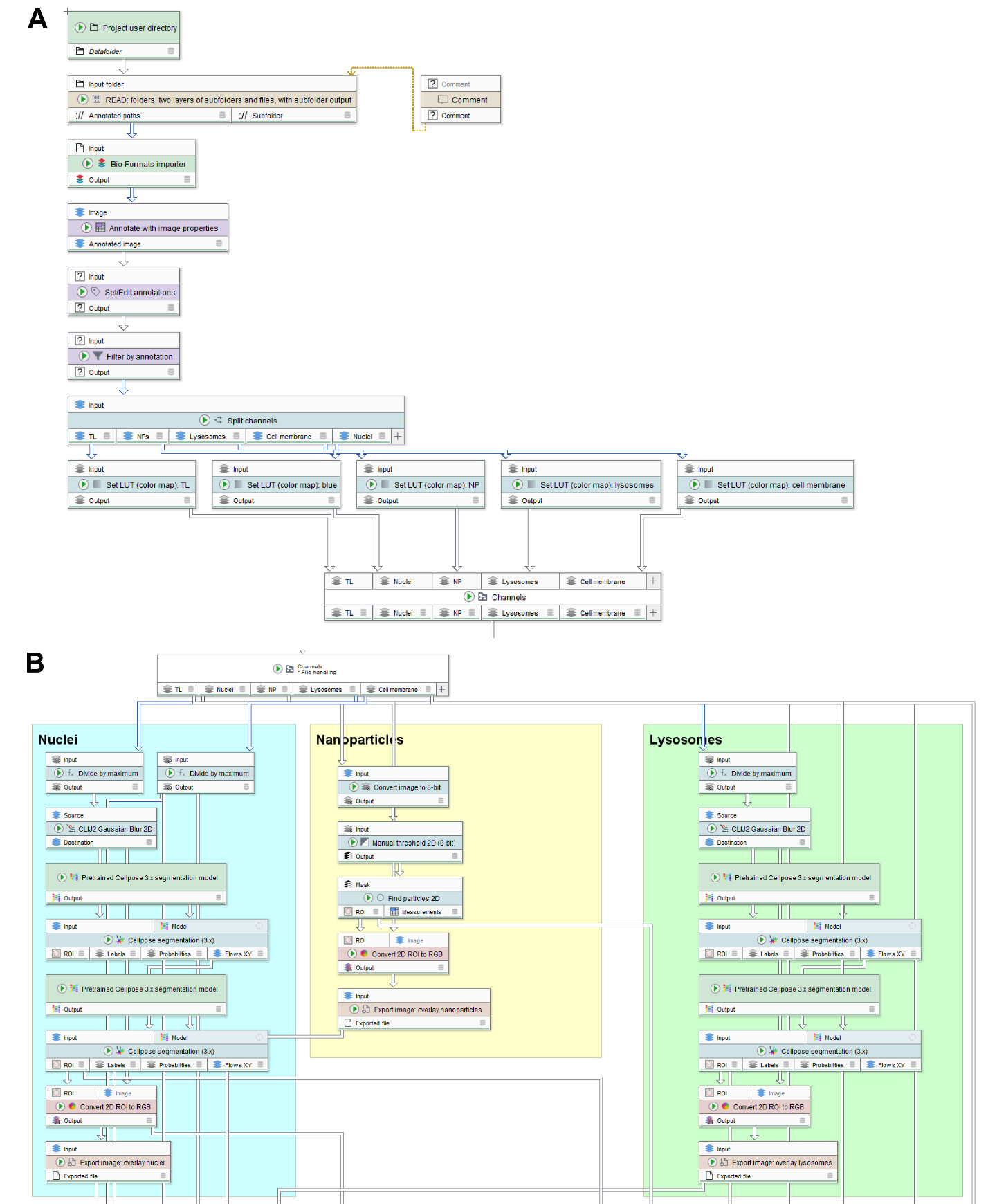


**Figure S6**: The detailed nodes structure of the JIPipe workflow. A) The compartment designed to read and annotate the image files. B) The nodes that were used to segment the four components as shown in Figure S5A.

## Synthesis and characterization of polymers

### Materials

L(-)-Lactide and glycolide (99.5%) were purchased from Corbion Biomaterials. Tin(II) 2-ethylhexanoate (Sn(Oct)_2_, 92.5-100.0%) and toluene (anhydrous, 99.8%) were supplied by Sigma-Aldrich. Sn(Oct)_2_ was dissolved in toluene and azeotropically distilled to remove residual water. The synthesis of PEtOx_1.8k_-OH with a degree of polymerization (DP) of 18 is described by Stafast *et al.*.^[6]^ All other chemicals were obtained from the usual suppliers and used without further purification.

### Instrumentation

^1^H NMR spectroscopy was performed at 298 K in deuterated dimethyl sulfoxide (DMSO-d_6_) using a 300 MHz Avance I spectrometer from Bruker, equipped with a dual proton (^1^H) and carbon (^13^C) sample head and an automatic BACS 120 sample changer (Bruker, Germany).

Size exclusion chromatography (SEC) was measured on an Agilent 1200 series system, equipped with a PSS degasser, a G1310A pump, a G1329A auto sampler and a Techlab oven (40 °C). A G7162A refractive index detector (RID) was utilized for data acquisition. A solution of 0.21% w/w LiCl in *N,N*-dimethylacetamide (DMAc) was used as eluent. The flow rate was adjusted to 1 mL min^-1^. A PSS Gram guard 30 Å and a PSS Gram guard 1,000 Å column were placed in series and served as a column set (10 μm particle size). The number averages of the molar mass (M_n_) as well as the dispersities (Ð) were determined using poly(styrene) (PS, Agilent Technologies, 400 to 1,000,000 g mol^-1^) as calibration standard.

### Synthesis of PEtOx_1.8k_-b-PLGA_16.8k_

The ring-opening polymerization was performed under inert conditions following a procedure published by Dirauf *et al.*.^[7]^

A preheated schlenk flask was used as a reaction vessel, which was cooled to room temperature (RT) under an argon atmosphere. 1,808 mg PEtOx_1.8k_-OH (DP = 18, 1.0 mmol), 6,313 mg L(-)-lactide (43.8 mmol) and 5,084 mg glycolide (43.8 mmol) were added into the flask. Subsequently, the mixture was heated to 130 °C. 2.35 mL of a stock solution of Sn(Oct)_2_ in anhydrous toluene (c = 172 mg mL^-1^, 1.0 mmol) were added to the melt to initiate the reaction, which was allowed to proceed for 45 min. After cooling to RT, the bulk was dissolved in 180 mL DMSO. For purification, the polymer was precipitated in deionized water. After filtration, the product was dried by lyophilization.

Yield: 13.39 g. Conversion L(-)-lactide: 95%. Conversion glycolide: quantitative. DP L(-)-lactide: 65. DP glycolide: 64.

^1^H NMR (300 MHz, DMSO-d_6_): *δ*= 0.85-1.05 (m, 54 H, CH_3_, PEtOx side chain); 1.29-1.62 (m, 127 H, CH_3_, lactate unit); 2.14-2.38 (m, 39 H, CH_2_, PEtOx side chain); 3.06-3.67 (m, 218 H, CH_2_, PEtOx backbone, water); 4.66-5.02 (m, 254 H, CH_2_, glycolate unit); 5.03-5.35 (m, 130 H, CH, lactate unit) ppm.

SEC (DMAc, 0.21% w/w LiCl; RID; PS calibration): M_n_ = 10,600 g mol^-1^; Ð = 2.54.


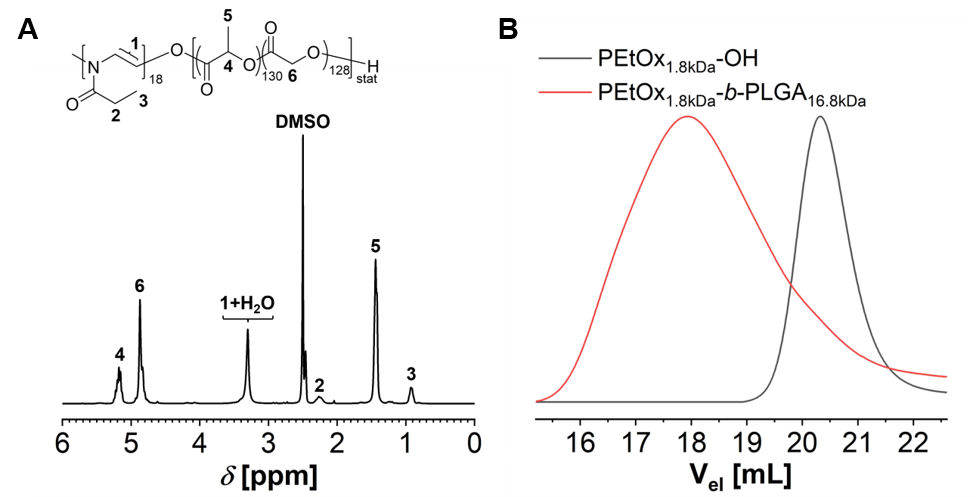


**Figure S7**: A) ^1^H NMR spectrum (300 MHz, DMSO-d_6_) of PEtOx_1.8k_-*b*-PLGA_16.8k_ and assignment of the signals to the schematic representation of the polymeric structure. B) Overlay of the SEC elugrams (DMAc, 0.21% w/w LiCl; RID) of PEtOx_1.8k_-*b*-PLGA_16.8k_ and the corresponding macroinitiator PEtOx_1.8k_-OH.

**References**

1. J. Drube; R. S. Haider; E. S. F. Matthees; M. Reichel; J. Zeiner; S. Fritzwanker; C. Ziegler; S. Barz; L. Klement; J. Filor; V. Weitzel; A. Kliewer; E. Miess-Tanneberg; E. Kostenis; S. Schulz; C. Hoffmann. GP CR kinase knockout cells reveal the impact of individual GRKs on arrestin binding and GPCR regulation. *Nat Commun* **2022,** *13* (1), 540, DOI: 10.1038/s41467-022-28152-8.

2. C. A. Schneider; W. S. Rasband; K. W. Eliceiri. NIH Image to ImageJ: 25 years of image analysis. *Nat Methods* **2012,** *9* (7), 671-5, DOI: 10.1038/nmeth.2089.

3. J. Schindelin; I. Arganda-Carreras; E. Frise; V. Kaynig; M. Longair; T. Pietzsch; S. Preibisch; C. Rueden; S. Saalfeld; B. Schmid; J. Y. Tinevez; D. J. White; V. Hartenstein; K. Eliceiri; P. Tomancak; A. Cardona. Fiji: an open-source platform for biological-image analysis. *Nat Methods* **2012,** *9* (7), 676-82, DOI: 10.1038/nmeth.2019.

4. R. Gerst; Z. Cseresnyes; M. T. Figge. JIPipe: visual batch processing for ImageJ. *Nat Methods* **2023,** *20* (2), 168-169, DOI: 10.1038/s41592-022-01744-4.

5. M. Pachitariu; C. Stringer. Cellpose 2.0: how to train your own model. *Nat Methods* **2022,** *19* (12), 1634-1641, DOI: 10.1038/s41592-022-01663-4.

6. L. M. Stafast; M. Swieczkowski; P. Poudel; N. Engel; C. Yin; K. Scheuer; C. Weber; F. H. Schacher; K. D. Jandt; U. S. Schubert. POxylated stereocomplexes from PEtOx-b-PLA diblock copolymers. *Eur Polym J* **2024,** *221*, 113545, DOI: 10.1016/j.eurpolymj.2024.113545.

7. M. Dirauf; C. Grune; C. Weber; U. S. Schubert; D. Fischer. Poly(ethylene glycol) or poly(2-ethyl-2-oxazoline) – A systematic comparison of PLGA nanoparticles from the bottom up. *Eur Polym J* **2020,** *134*, 109801, DOI: 10.1016/j.eurpolymj.2020.109801.
